# A Lipid Metabolism-Based Seven-Gene Signature Correlates with the Clinical Outcome of Lung Adenocarcinoma

**DOI:** 10.1155/2022/9913206

**Published:** 2022-02-11

**Authors:** Tianqi Li, Jiquan Chen, Jun Liu, Qingjie Chen, Wei Nie, Mi-Die Xu

**Affiliations:** ^1^Department of Pathology, Fudan University Shanghai Cancer Center, Shanghai 200032, China; ^2^Department of Oncology, Shanghai Medical College, Fudan University, Shanghai 200032, China; ^3^Institute of Pathology, Fudan University, Shanghai 200032, China; ^4^Department of Respiratory Medicine, The Third Affiliated Hospital of Naval Medical University, Shanghai 200438, China; ^5^Department of Anesthesiology, Shanghai Pulmonary Hospital, Tongji University, Shanghai 200433, China; ^6^Department of Nuclear Medicine, the First Affiliated Hospital of Nanchang University, Nanchang 200433, China; ^7^Department of Pulmonary Medicine, Shanghai Chest Hospital, Shanghai Jiaotong University, Shanghai, China

## Abstract

**Background:**

Herein, we tried to develop a prognostic prediction model for patients with LUAD based on the expression profiles of lipid metabolism-related genes (LMRGs).

**Methods:**

Molecular subtypes were identified by non-negative matrix factorization (NMF) clustering. The overall survival (OS) predictive gene signature was developed and validated internally and externally based on online data sets. Time-dependent receiver operating characteristic (ROC) curve, Kaplan–Meier curve, nomogram, restricted mean survival time (EMST), and decision curve analysis (DCA) were used to assess the performance of the gene signature.

**Results:**

We identified three molecular subtypes in LUAD with distinct characteristics on immune cells infiltration and clinical outcomes. Moreover, we confirmed a seven-gene signature as an independent prognostic factor for patients with LUAD. Calibration and DCA analysis plots indicated the excellent predictive performance of the prognostic nomogram constructed based on the gene signature. In addition, the nomogram showed higher robustness and clinical usability compared with four previously reported prognostic gene signatures.

**Conclusions:**

Findings in the present study shed new light on the characteristics of lipid metabolism within LUAD, and the established seven-gene signature can be utilized as a new prognostic marker for predicting survival in patients with LUAD.

## 1. Introduction

Globally, lung cancer is the leading cause of cancer death worldwide with a five-year survival rate of advanced-stage patients less than 5% [1, 2]. As the major histological subtype of lung cancer (approx. 85% cases), lung adenocarcinoma (LUAD) results in the death of most patients from local recurrence or distant metastasis [2]. The prognosis of advanced LUAD is still not satisfactory, and treatment options are limited [3]. In order to anticipate the natural history of the disease in individual patients, it is mandatory that the nature of LUAD in each patient should be clearly understood since only individually tailored molecular profiles and markers could spare the patients from undergoing a potentially more harmful, aggressive chemical therapy or even leave them untreated. Therefore, there is an increasing interest in the molecular characterization of LUAD allowing prognosticate overall patient survival.

The interaction of tumor cells and lipid metabolism is supposed to play an important role in LUAD progression [4, 5]. Although metabolism reprogramming was presumed to be one of the hallmarks of tumorigenesis, molecular characteristics of lipid metabolic disorder within the tumor microenvironment are incompletely understood. Almost nothing is known about the precise role and underlying mechanism of lipid metabolism-related genes (LMRGs) and their gene expression profiles in primary resected LUAD, not yet anything known related to the prognostic distinctions of LUAD.

The current study aimed at the discovery of a new prognostic signature in patients with LUAD using Cox and the least absolute shrinkage and selection operator (LASSO) regression models to analyze the expression profile of LMRGs using The Cancer Genome Atlas (TCGA) LUAD data set. Based on LMRG expression data from public databases in TCGA, we constructed molecular subtypes with distinct different immune characteristics and clinical outcomes. Then, we developed a seven-LMRG signature for assessing the prognosis of patients with LUAD and further validated it in TCGA, GSE31210, and GSE50081 data sets. This signature was tightly associated with patients' outcomes and could serve as an independent pathological factor.

## 2. Methods and Materials

### 2.1. Patients and Data Sets

The RNA‐seq data and clinical information of 594 LUAD samples were downloaded from the TCGA database (http://www.cancer.gov/about-nci/organization/ccg/research/structural-genomics/TCGA). TCGA LUAD data set was preprocessed with the criteria as follows: (1) excluded samples without clinical data and OS < 30 days; (2) excluded data of normal esophagus tissue sample; (3) excluded genes with FPKM = 0 in 50% of samples; and (4) included the expression profile of genes related to lipid metabolism. Finally, 490 samples from TCGA LUAD data set were enrolled for subsequent analysis. Two data sets, which contains gene expression profiles of LUAD samples in the GEO database, GSE31210 (contains 226 LUAD samples) and GSE50081 (contains 129 LUAD samples), were downloaded from NCBI (http://www.ncbi.nlm.nih.gov/geo/). Both data sets were preprocessed with the criteria as follows: (1) excluded normal tissue sample data; (2) converted OS data from year or month to days; (3) transformed gene probes to the human gene SYMBOL using the bioconductor package of R, those matched to multiple genes were removed, and if multiple probes were matched one gene, the median was selected as the expression profile of this gene; and (4) microarray data was normalized from Affymetrix platform by using robust multiarray average method [6]. The clinicopathological characteristics of patients from these three data sets after preprocessing are summarized in Table 1.

For the TCGA LUAD data set, 90% of them was randomly divided into training cohorts (*n* = 441), and the entire data set was selected as a validation cohort. GSE31210 and GSE50081 data sets were used as external validation cohorts. For real-time quantitative PCR (RT-qPCR) and immunochemistry (IHC) analysis, 10 NSCLC patients undergoing resection were enrolled in this study from the biobank of FUSCC. Surgically excised tumors used for qRT-PCR were immediately immersed in liquid nitrogen and stored at −80°C. At the same time, formalin-fixed paraffin-embedded (FFPE) tissues were also obtained from the same patient's tissues for IHC.

This study was approved by the Institutional Review Boards of Fudan University Shanghai Cancer Center (FUSCC).

### 2.2. Identification of Molecular Subtypes Based on Lipid Metabolic Genes

A total of 776 genes related to lipid metabolism from six lipid metabolism-related gene sets (Supplementary Table 1) were collected from the Molecular Signature Database v7.0 (MSigDB). Among them, 3 genes were not offered in the TCGA LUAD data set, and 30 genes with FPKM = 0 in more than half of the samples were also excluded. Finally, 743 genes were enrolled for subsequent analysis. Prognostic genes were detected by univariate Cox regression survival analysis using the R package survival coxph function, and log rank *P* < 0.05 was selected as the threshold. The molecular subtypes were identified based on these prognostic genes using the non-negative matrix factorization (NMF) method, and the optimal number of subtypes were determined according to indicators including cophenetic correlation, residual sum of squares (RSS), dispersion, and silhouette. Cophenetic correlation range between 0 and 1, it positively reflect the stability of the cluster obtained from NMF [7]. The silhouette coefficient can be used to select the appropriate number of clusters. According to the line chart, the point with the largest coefficient change range can be found intuitively, and the point with the largest distortion range reflects the best number of clusters [8]. A residual sum of squares (RSS) is a statistical technique used to measure the amount of variance in a data set that is not explained by a regression model [9]. Thus, the RSS value negatively reflects the clustering performance of the model.

### 2.3. Analysis of Gene Expression Profile and Immune Score among Molecular Subtypes

DESeq2 was used to calculate differentially expressed genes (DEGs) among each cluster (FDR <0.05 and |log2FC| > 1). Weighted gene correlation network analysis (WGCNA) coexpression algorithm was used for detecting coexpressed genes and modules by the R package WGCNA [10]. To improve the accuracy of network construction, the transcripts per kilobase of exon model per million mapped reads (TPM) profile of genes were extracted from the TCGA LUAD data sets, and hierarchical cluster analysis was performed on the 490 samples first to remove the outlier samples. Second, the distance between each gene was calculated using the Pearson correlation coefficient; a weighted coexpression network was constructed using the R package WGCNA; and coexpression modules were screened by setting the soft threshold power *β* as 7. Third, the topology overlap matrix (TOM) was then constructed from the adjacency matrix to avoid the influence of noise and spurious associations. On the basis of TOM, average-linkage hierarchical clustering using the dynamic shear tree method was subsequently conducted to define coexpression modules, and the minimum gene size of each module was set as 30. The feature vector values (eigengenes) of each module were calculated in turn to explore the relationship among modules, and then modules with highly correlated eigengenes were merged into a new module by performing cluster analysis with the following threshold: height = 0.25, DeepSplit = 2, and minModuleSize = 30. In order to identify the modules of interest, the correlation between each coexpression module and patients' clinical features as well as cluster subtypes was further evaluated. Modules with significant correlation with the lipid-metabolism subtypes of LUAD patients were defined as key modules for the subsequent selection of hub genes (Spearman correlation coefficient >0.4; *P* < 0.05).

Tumor immune estimation resource (TIMER) tool [11] was used to compare the enumeration of six tumor-infiltration immune cell types (including B cell, CD4^+^T cell, CD8^+^T cell, neutrophil, macrophage, and dendritic cell) of each LUAD sample in the tumor microenvironment (Supplementary Table 2). Next, the difference on the ImmuneScore and StromalScore, which represent the relative proportion of immune cells and stromal cells in tumor tissues, among each molecular subtype was calculated by using the R package Estimation of STromal and Immune cells in MAlignant Tumours using Expression data (ESTIMATE) [12]. The ESTIMATEScore, which refers to the purity of tumor tissues, is the sum of ImmuneScore and StromalScore.

### 2.4. Construction of Lipid Metabolism-Related Prognostic Gene Signature

Coexpression genes in the training set were detected by univariate Cox regression survival analysis, and log rank *P* < 0.01 was selected as the threshold. To narrow the gene range and maximize the accuracy, Least absolute shrinkage and selection operator (LASSO) Cox regression analysis [13], a method screening signatures with generally effective prognostication performance by performing automatic feature selection, was performed by using the glmnet package of R to identify the prognostic gene. And optimal genes were evaluated by tenfold cross-validation.

Multivariate Cox regression survival analysis was performed to construct the prognostic risk model. The risk score for each patient of the training set was calculated with the linear combinational of the signature gene expression weighted by their regression coefficients. Risk score = (expr_gene1_ × coefficient_gene1_) + (expr_gene2_ × coefficient_gene2_) + … + (expr_genen_ × coefficient_genen_). Receiver operating characteristics (ROC) curves, carried out by using the R package timeROC, were used to analyze the risk score of each sample, and samples were set as a high- or low-risk group by setting the threshold as 0.

### 2.5. Bioinformatic Analysis

Pathway enrichment analysis of differentially coexpressed genes was performed through the R package WebGestaltR (the threshold FDR <0.05). Single-sample gene set enrichment analysis (ssGSEA) was applied for identifying the relationship between the risk scores of different samples and biological functions using the R package GSVA. The classical gene sets of Kyoto Encyclopedia of Genes and Genomes (KEGG) pathways (c2.cp.kegg.v7.0.symbols) were considered to decipher the phenotype. For each analytical pathway, the enrichment score (ES) and the significance of ES were calculated, and the normalized enrichment score (NES) and false discovery rate (FDR) were further calculated to examine functional enrichment results. An FDR cutoff value of 0.05 was considered in this test.

### 2.6. Statistical Analysis

Kaplan–Meier curves were applied to assess the difference in OS between different groups. Multivariate Cox regression analyses were performed to assess the independent prognostic factors. Decision curve analysis (DCA), which can evaluate predictive models from the perspective of clinical consequences [14], was performed in the entire cohort to test the clinical usefulness of the nomogram in comparison with the gene signature and clinicopathological parameters. All statistical analyses were using R 3.6.0 (https://mirrors.tuna.tsinghua.edu.cn/CRAN/) with default software parameters. *P* value <0.05 was considered significant statistically.

*Immunohistochemistry Analysis*. Formalin-fixed and paraffin-embedded specimens were cut into 5 mm thick sections and mounted on glass slides. IHC was carried out as previously reported [15]. CD4 antibody and CD8 antibody were purchased from Abcam. DAB reagent was purchased from Fansbio (Guangzhou, China).

### 2.7. RT-PCR

Total RNA was harvested from LUAD tissue using Trizol and reverse-transcribed into cDNA using PrimeScript RT reagent kit (with gDNA Eraser) (Takara). The cDNA was amplified using the SYBR qPCR Master Mix (Takara) on a real-time PCR system (LightCycler480). Primer sequences are shown in Table 2. All primers were synthesized by Sangon Biotech Co. Ltd. (Shanghai). RT-PCR was carried out as previously reported [15].

## 3. Result

### 3.1. Identification of Molecular Subtypes in LUAD

**Figure 1:**
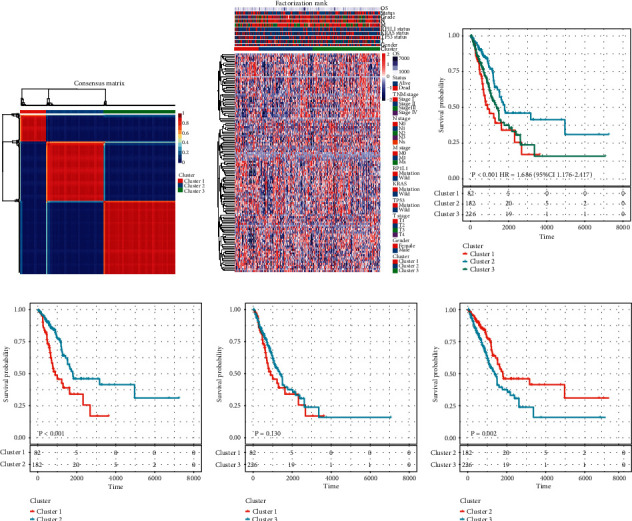
Identification of molecular subtypes in LUAD: (a) consensus map of NMF clustering, (b) heat map of the expression profile of 740 lipid-metabolism-related genes (LMRGs) and the distribution of clinicopathological parameters in all three subtypes, (c) Kaplan–Meier curves showed the overall survival (OS) curve of the three subtypes, and (d) Kaplan–Meier curves showed the overall survival (OS) curve of every two subtypes.

By univariate Cox regression survival analysis, 126 lipid metabolism-related genes were identified correlated with the overall survival (OS) of patients with LUAD in the TCGA data set (Supplementary Table 3). Then, by using non-negative matrix factorization (NMF) method, three molecular subtypes (Cluster 1 (*n* = 82), Cluster 2 (*n* = 182), and cluster 3 (*n* = 226)) were constructed based on these prognostic genes (Figure 1(a); Supplementary Figure 1(a)). Gene expression profile and the distribution of clinicopathological parameters in each subtype were shown in Figure 1(b). We further analyzed the relationship between the molecular subtype and clinicopathological features of LUAD patients. It was observed that most of the patients at the M1 stage were divided into Cluster 1, which had worse survival. The Cluster 2 with the best outcomes inversely showed a trend of younger age and early TNM stage and exhibited wild TP53 and RP1L1 status (Supplementary Figure 1(b)). In addition, the differences in immune characteristics among the three subtypes were analyzed. Cluster 1 showed the lowest proportions of B cell, CD4^+^T cell, CD8^+^T cell, neutrophil, macrophage, and dendritic cell (DC) than the other two subtypes (Supplementary Figure 1(c)). The calculated ImmuneScore, StromalScore, and ESTIMATEScore were also remarkably lower in Cluster 1, which represented less immune and stromal cell components in tumor microenvironment (TME) and lower tumor priority for the samples in the other two subtypes (Supplementary Figure 1(d)); in contrast, Cluster 2 showed the highest ImmuneScore, which suggests that patients in Clusters 1 and 2 have distinct immune and stroma characteristics. Furthermore, Kaplan–Meier method with log-rank tests was applied to explore the difference in prognosis among the three molecular subtypes in LUAD. Although it seems that patients in Cluster 1 confer the worst OS (Figure 1(c)), there was no significant difference between Clusters 1 and 3 (Figure 1(d)). However, compared with Cluster 1 (*P* < 0.001) and Cluster 2 (*P* = 0.002), patients in Cluster 2 showed the longest OS time (Figure 1(d)).

### 3.2. WGCNA Coexpression Analysis

**Figure 2:**
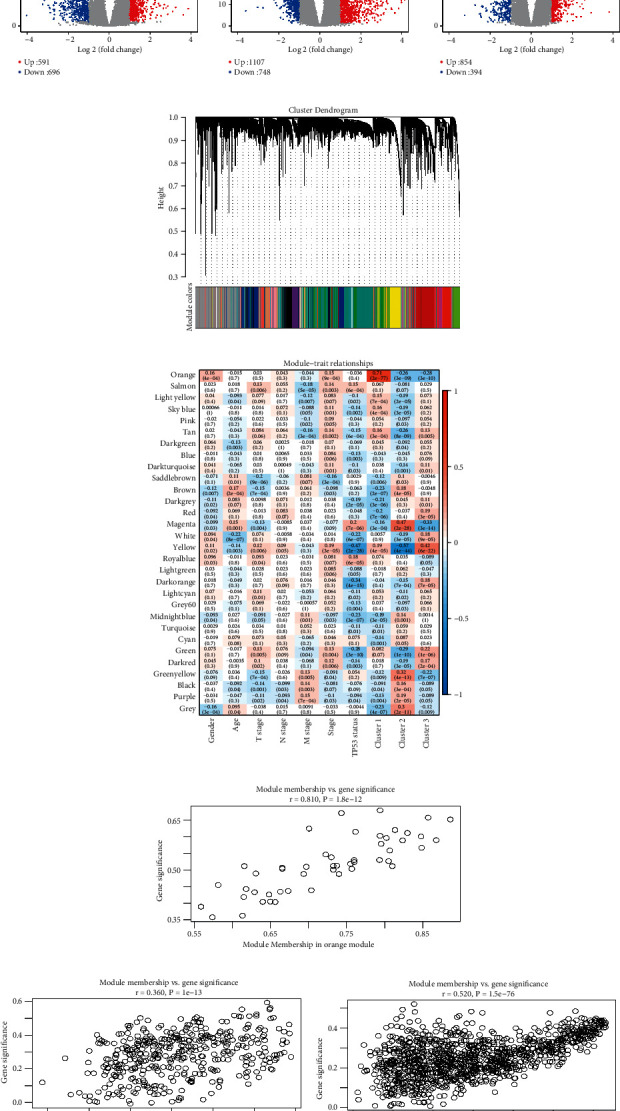
WGCNA coexpression analysis: (a) volcano map of differentially expressed genes (DEG) between each cluster and the other subtypes, (b) gene dendrogram and module colors, and (c) relationship between the 30 modules and the clinical phenotypes and molecular subtypes. The correlation of orange module with Cluster 1 (d), magenta module with Cluster 2 (e), and yellow module with Cluster 3 (f) in the TCGA data set.

Genes differentially expressed among each molecular subtype were calculated. There are 1,287 differentially expressed genes (DEGs; 591 up-regulated and 696 down-regulated; Figures 2(a)) in Cluster 1 compared with the other 2 subtypes, 1,855 DEGs (1,107 up-regulated and 748 down-regulated; Figures 2(a)) between Cluster 2 and other two subtypes, and 1,748 DEGs (854 up-regulated and 894 down-regulated; Figures 2(a)) between Cluster 3 and other two subtypes. By hierarchical clustering the expression profiles of the prognostic gene, we found no samples with outliers in the TCGA LUAD data sets (Supplementary Figure 2(a)). To ensure that the coexpression network constructed by WGCNA is scale-free, we set the soft threshold as 7 (Supplementary Figures 2(b)–2(c)). Based on the expression of LMRGs in the TCGA LUAD data set, a total of 30 coexpression modules were obtained after module fusion (Figure 2(b); grey modules represent gene sets that could not be merged). Moreover, by analyzing the correlation of the module and genes in the module with phenotypes (Figure 2(c)), we found that orange (contains 49 genes) module correlated with Cluster 1, magenta (contains 404 genes) module correlated with Cluster 2, and yellow (contains 1090 genes) module correlated with Cluster 3 (Figures 2(d)–2(f)). Further analysis of these 4,390 DEGs revealed that there were 486 overlapping DEGs among all three subtypes (Supplementary Figure 2(d)).

To further investigate the biological functions of these 486 overlapping DEGs, gene ontology (GO) and pathway analysis were performed. KEGG analysis has identified 27 significant pathways, such as p53 signaling pathway and complement and coagulation cascades (Supplementary Figure 2(e)). GO analysis results showed that the DEGs were clustered in 93 significant cellular component (CC) categories with kinesin complex ranked as the most significant CC category (Supplementary Figure 2(f)); 87 significant molecular function (MF) categories with motor activity ranked as the most significant MF category (Supplementary Figure 2(g)); and 620 significant biological process (BP) categories with the regulation of mitotic cell cycle phase transition ranked as the most significant BP category (Supplementary Figure 2(h)).

### 3.3. Construction of Prognosis Risk Model Based on Differential Coexpression Genes

To identify novel genetic biomarkers associated with the clinical outcome of patients with LUAD, univariate Cox proportional hazard regression was applied to these 486 DEGs. And then 186 genes significantly correlated to OS (*P* < 0.01; Supplementary Table 4) were entered into dimensional-reduction analysis by performing LASSO regression analysis, and seven prognostic DEGs (including CHRDL1, GAPDH, GNPNAT1, HTATIP2, MFI2, PKP2, and RGS20) were confirmed with tenfold cross-validation and the minimized error rate *λ* = 0.059 (Supplementary Figures 3(a)–3(b)). By applying Kaplan–Meier analysis, we confirmed that all these seven genes were significantly associated with the OS of patients in the training set (Supplementary Figure 3(c)). Among them, CHRDL1 (*P* < 0.001) showed a significant negative correlation with OS, while the other six genes were positively correlated to OS (all *P* < 0.001; Supplementary Figures 3(d)–3(i)). The final seven-gene signature was calculated using multivariate Cox survival analysis (Table 3), and a gene-based prognostic model was established to evaluate the survival risk of each patient as follows: RiskScore = −0.0103 ∗ exp^CHRDL1^ + 0.0001 ∗ exp^GAPDH^ + 0.0105 ∗ exp^GNPNAT1^ + 0.0039 ∗ exp^HTATIP2^ + 0.0064 ∗ exp^MFI2^ + 0.0085 ∗ exp^PKP2^ + 0.0284 ∗ exp^RGS20^.

**Figure 3:**
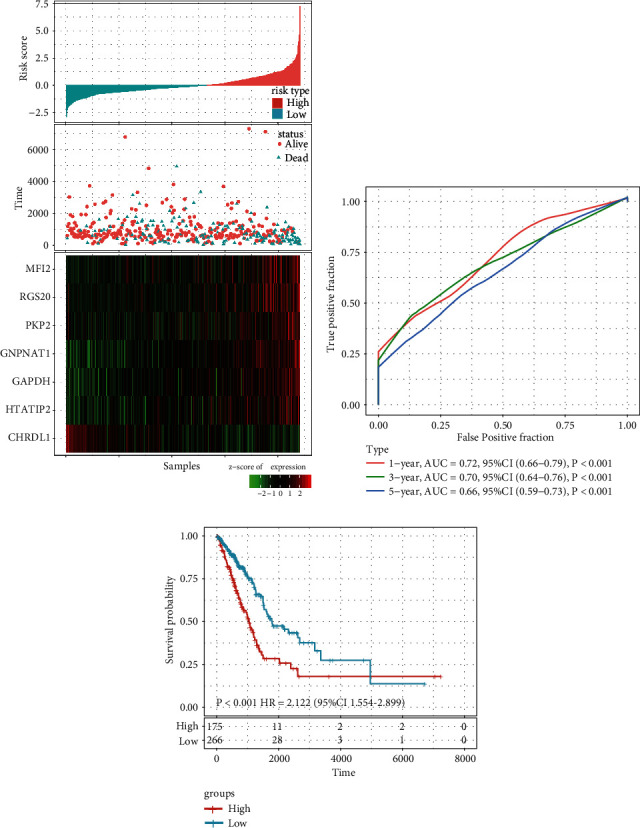
Evaluation of the performance of the seven-gene signature in the training data set: (a) risk score, survival time, survival status, and expression of the seven-gene signature in the training set; (b) ROC curve of the seven-gene signature for 1-, 3-, and 5-year survival in the training set; and (c) Kaplan–Meier survival analysis of overall survival for high- or low-risk group patients in the training set. ROC, receiver operating characteristic; AUC, area under the curve; HR, hazard ratio; and CI, confidence interval.

Based on the risk score formula and the cut-off value of normalized risk score (Z-score = 0), patients were divided into a high- or low-risk group (Figure 3(a)). And a heatmap showing the expression profile of the eight genes illustrated that as the risk score of patients increased, the expression of prognosis-risky genes (GAPDH, GNPNAT1, HTATIP2, MFI2, PKP2, and RGS20) were distinctly up-regulated; in contrast, the expression of prognosis-protective gene CHRDL1 was down-regulated. ROC curve showed that the accuracy of the prognostic seven-gene signature for 1-, 3-, and 5-year survival was 0.72, 0.70, and 0.66, respectively (Figure 3(b)). Finally, we divided the samples with Z-score-based RiskScore greater than zero into the high-risk group and the samples with less than zero into the low-risk group. Kaplan–Meier curve analysis revealed that the OS time of patients in the high-risk group was significantly shorter than that in the low-risk group (HR = 2.122; 95% CI: 1.554–2.899; *P* < 0.001; Figure 3(c)).

### 3.4. Validation of the Seven-Gene Signature in the Entire TCGA Data Set and Two GEO LUAD Data Sets

**Figure 4:**
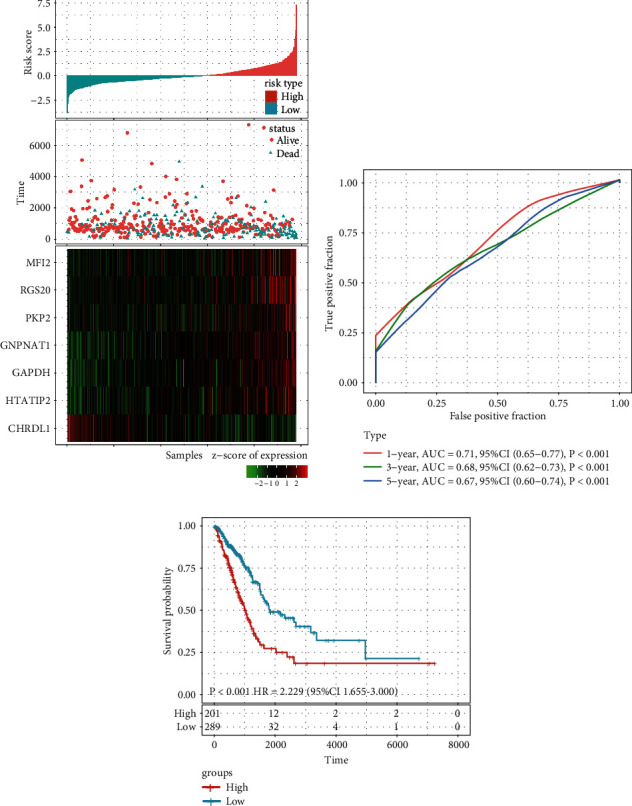
Internal validation of the seven-gene signature's robustness in the entire TCGA cohort: (a) risk score, survival time, survival status, and expression of the seven-gene signature in the internal validation set; (b) ROC curve of the seven-gene signature for 1-, 3-, and 5-year survival in the internal validation set; and (c) Kaplan–Meier survival analysis of overall survival for high-risk or low-risk group patients in the internal validation set. ROC, receiver operating characteristic; AUC, area under the curve; HR, hazard ratio; and CI, confidence interval.

The entire TCGA LUAD data set (*n* = 490) was used for internal validation, and the risk score of each sample was calculated, which showed that the association between the gene expression and risk score was consistent with the training set (Figure 4(a)). The ROC curve displayed that the accuracy of the prognostic eight-gene signature for 1-, 3-, and 5-year survival was 0.71, 0.68, and 0.67, respectively (Figure 4(b)). Patients in the internal validation data set were classified into high- and low-risk groups with the same cutoff as used in the training set. As expected, patients in the validation set with high risk scores had shorter OS than those with low risk scores (HR = 2.229; 95% CI: 1.655–3.000; *P* < 0.001; Figure 4(c)).

**Figure 5:**
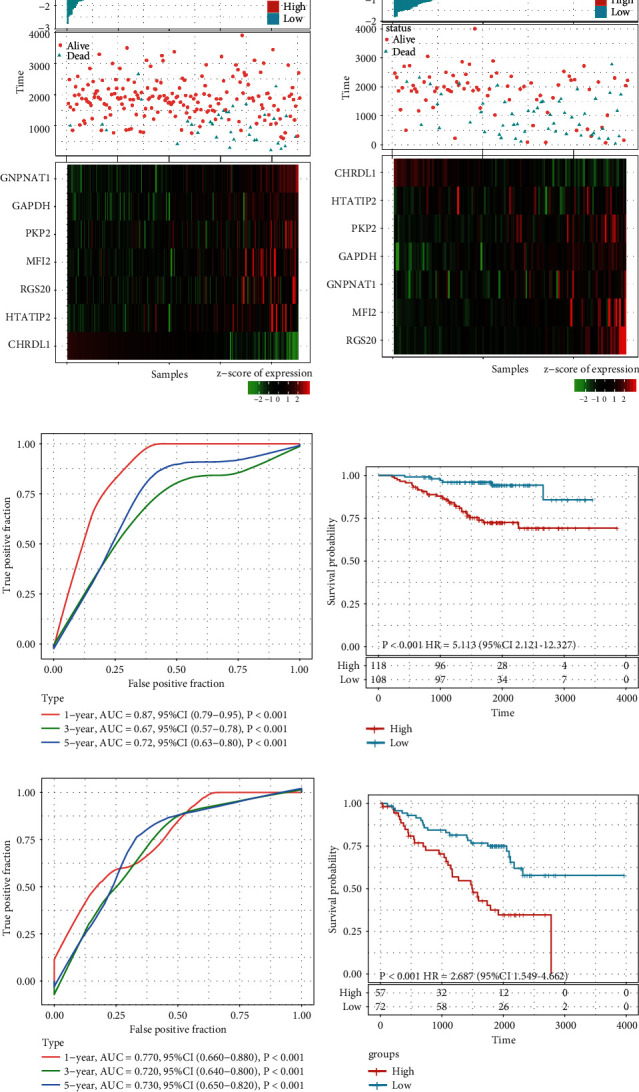
External validation of the seven-gene signature's robustness in the GSE31210 and GSE50081 cohorts. Risk score, survival time, survival status, and expression of the seven-gene signature in the GSE31210 (a) and GSE50081 (b) cohorts, respectively. (c) ROC curve of the seven-gene signature for 1-, 3-, and 5-year survival in the GSE31210 cohort. (d) Kaplan–Meier survival curve based on the seven-gene signature in the GSE31210 cohorts. (e) ROC curve of the seven-gene signature for 1-, 3-, and 5-year survival in the GSE50081 cohort. (f) Kaplan–Meier survival curve based on the seven-gene signature in the GSE31210 cohorts. ROC, receiver operating characteristic; AUC, area under the curve; HR, hazard ratio; and CI, confidence interval.

Subsequently, the prognostication efficiency of our seven-gene signature was also calculated in the two external validation data sets GSE31210 and GSE50081. The results showed that the association between the gene expression and risk score was consistent with that in the training and internal validation set (Figure 5(a) and 5(b)). In the GSE31210 data set, The ROC curve displayed that the accuracy of the prognostic seven-gene signature for 1-, 3-, and 5-year survival was 0.87, 0.67, and 0.72, respectively (Figure 5(c)). As expected, patients in the GSE31210 data set with high risk scores had shorter OS than those with low risk scores (HR = 5.113; 95% CI: 2.121–12.327; *P* < 0.001; Figure 5(d)). In the GSE50081 data set, the ROC curve displayed that the accuracy of the prognostic seven-gene signature for 1-, 3-, and 5-year survival was 0.77, 0.72, and 0.73, respectively (Figure 5(e)). As expected, patients in the GSE50081 data set with high risk scores had shorter OS than those with low risk scores (HR = 2.687; 95% CI: 1.549–4.662; *P* < 0.001; Figure 5(f)). Therefore, the seven-gene signature exhibited steady effective prognostic classification performance in the internal and two external validation sets.

### 3.5. Univariate and Multivariate Cox Regression Analyses of the Seven-Gene Signature

In order to identify the independence of the seven-gene signature model in clinical application, univariate and multivariate Cox regression analysis was used to systematically analyze the clinical outcome of patients in the entire TCGA LUAD data set. Univariate analysis of survival revealed that the seven-gene signature (*P* < 0.001), age (*P* = 0.038), N stage (*P* < 0.001), M stage (*P* < 0.001), and TP53 mutation status (*P* < 0.001) were prognostic indicators of OS (Supplementary Figure 4(a)). However, multivariate Cox regression analysis showed that only seven-gene signature (*P* < 0.001) in addition to TNM stage (*P* = 0.001) were independent risk factors of OS (Supplementary Figure 4(b)). Overall, these results suggest that the seven-gene signature is a potential independent prognostic factor for LUAD.

### 3.6. The Performance of the Seven-Gene Signature in Comparison to Previous Signatures in TCGA LUAD Data Set

**Figure 6:**
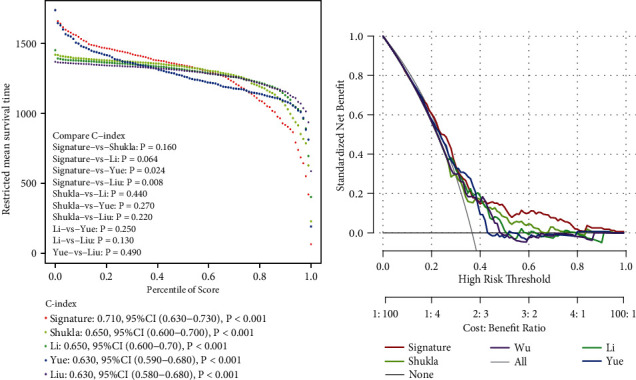
The performance of the seven-gene signature in comparison to previous signatures in the TCGA LUAD data set: (a) restricted mean survival time (RMST) curve developed by integrating the signatures and (b) decision curve analysis (DCA) for the integrating the signatures.

To assess the predictive power of the seven-gene signature, four published risk models for OS of patients with LUAD: a three-gene signature developed by Shukla et al. [16], an eight-gene signature developed by Li et al. [17], a three-gene signature developed by Yue et al. [18], and a three-gene signature developed by Liu et al. [19] were enrolled for comparison. To improve the comparability of the models, the risk score of each LUAD sample in the TCGA cohort was calculated according to the corresponding genes in all four models by applying the same method being reported [20–22]. Kaplan–Meier curve analysis revealed that all four modules showed significant prognostic value in predicting OS (all *P* < 0.001; Supplementary Figures 5(a)–5(d)). The ROC of each model was evaluated, and the area under the curve (AUC) of all three models were larger than 0.6 (Supplementary Figures 5(a)–5(d)), while only the AUC of 2-year OS in Shukla's three-gene signature and the AUC of 1-year OS in Li's eight-gene signature were larger than 0.7 (Supplementary Figures 5(a) and 5(b)). Restricted mean survival time (RMST) was applied to calculate and compare the C-index of all signatures. Although the C-index of our seven-gene signature was only significantly higher than that of Yue's module (*P* = 0.024) and Liu's module (*P* = 0.008), our seven-gene signature showed highest C-index (0.710; Figure 6(a)). We also applied a DCA to evaluate our seven-gene signature with these four signatures, in which the net benefit together with a broader range of threshold probability of our seven-gene signature ranked as the highest one (Figure 6(b)), indicating that the seven-gene signature in the present study exhibited a best predictive performance. Taken together, these results suggest that this seven-gene signature is more suitable for predicting the prognosis of patients with LUAD in clinical practice.

### 3.7. GSEA Analysis of Enriched Pathway Based on Risk Score

**Figure 7:**
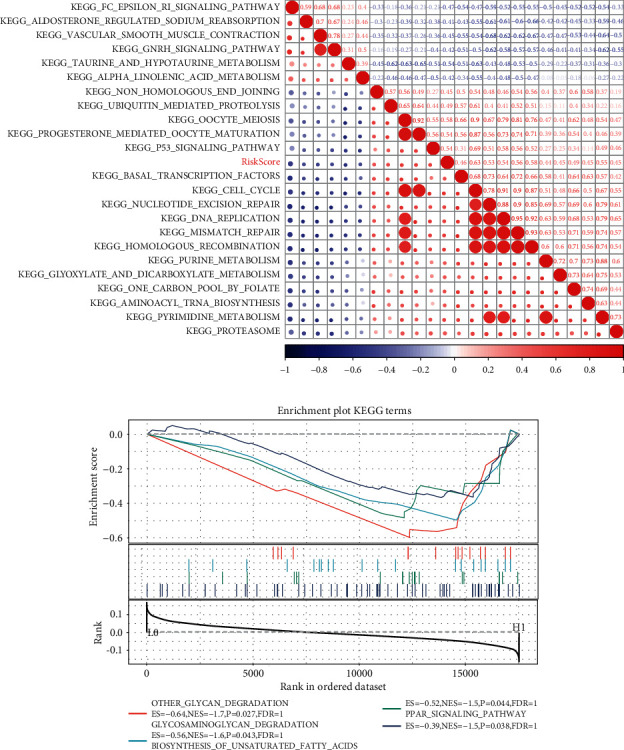
ssGSEA result according to the risk score of LUAD samples in the TCGA data set: (a) clustering of KEGG pathways correlated with RiskScore, with correlation coefficients greater than 0.40, and (b) enrichment pathways that were significantly correlated in the high-risk groups.

ssGSEA was performed to determine the potential related pathways according to patients' prognostic risk in the TCGA training data set, and pathways with Pearson correlation coefficient >0.4 were derived. As shown in Figure 7(a), a total of 23 pathways were identified, and most of the pathways were positively correlated with samples' risk score (red color). By dividing samples into high- and low-risk groups based on whether the risk score is greater than 0 and analyzing the enriched pathway in both groups by using GSEA, we found that four pathways were significantly enriched in the high-risk group: other glycan degradation, glycosaminoglycan degradation, biosynthesis of unsaturated fatty acids, and PPAR signaling pathway (*P* < 0.05; Figure 7(b)). Thus, the seven-gene signature may involve in the development and progression of LUAD by participating in these pathways.

### 3.8. Detection of Prognostic Gene Expression in Lung Adenocarcinoma Tissue

**Figure 8:**
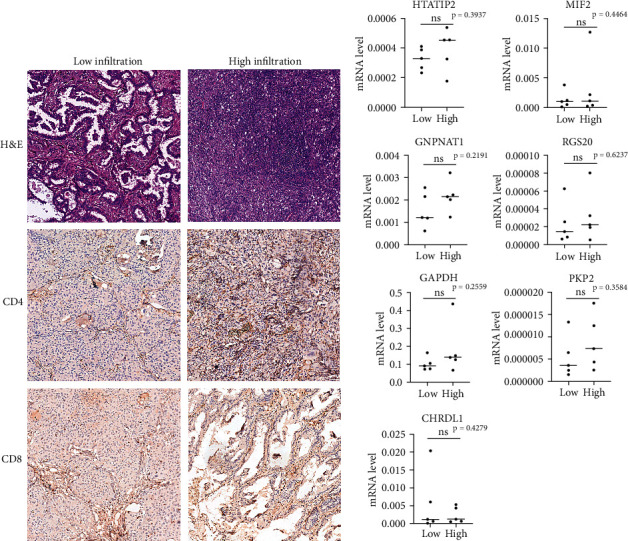
H&E staining, IHC staining, and mRNA levels of LUAD tissue: (a) representative H&E staining, CD4, and CD8 IHC staining result of LUAD tissue and (b) mRNA levels of GAPDH, GNPNAT1, HTATIP2, MFI2, PKP2, RGS20, and CHRDL1 in LUAD measured by RT-qPCR.

We selected ten patient tissues who underwent surgical treatment in the FUSCC in 2019 for the determination of the expression level of these seven prognostic genes in LUAD. According to the results of the H&E staining of tumor tissues and the immunohistochemical staining of CD4 and CD8 cells in tumor tissues, the tissues were divided into two groups: the low-immune infiltration group and the high-immune infiltration group (Figure 8(a)). Subsequently, we applied RT-PCR to detect the expression level of GAPDH, GNPNAT1, HTATIP2, MFI2, PKP2, RGS20, and CHRDL1 in these 10 tumor tissues. As shown in Figure 8(b), the mRNA level of GAPDH, GNPNAT1, HTATIP2, MFI2, PKP2, and RGS2 showed higher expression tendency in the high-immune infiltration group than the low-immune infiltration group, whereas the expression level of CHRDL1 showed a lower expression tendency in the high-immune infiltration group than the low-immune infiltration group.

## 4. Discussion

Cumulative evidences have been yielded in the field of lipid metabolism within lung cancer cells [23]. Under abnormalities in vessels structure attributed to limited nutrient supply and hypoxia, lung cancer cells present multiple metabolic alterations in order to support cell growth, including aerobic glycolysis and de novo lipogenesis [24]. De novo lipogenesis provides intermediate materials to support the phospholipid and triglyceride synthesis, lipid modification of proteins, and fatty acid *ß*-oxidation in tumor cells, including fatty acid synthase (FAS), stearoyl CoA desaturase 1 (SCD1), adenosine triphosphate-binding cassette family (ABC family), ATP citrate lyase (ACLY), and fatty acids and thus augments the activation of growth-promoting pathways [23]. Lung cancer cells have accelerated lipid metabolism (mainly de novo lipogenesis) than normal lung respiratory epithelium [25]. Therefore, lipid metabolism alteration may be a potential indicator of tumor malignancy and thus prognosis.

In order to better understand the biological characteristics of LUAD, we classified LUAD into three subtypes based on a comprehensive analysis of LMRG expression profiles in the TCGA LUAD data set. We demonstrated that the Cluster 1 subtype is associated with the lowest infiltration of immune cells, decreased immune scores, stromal scores, and tumor purity. This immune-related difference in LMRG-based molecular subtype may reflect the effects of lipid metabolism on tumor immune microenvironment. It has been reported that the lipid levels in DCs can influence the ability of DCs to process antigen [26]. In addition, immune cells can interact with various classes of lipids, and altering lipid metabolism is capable of controlling the activation, differentiation, plasticity, and function of immune cells [27]. To some extent, our data indicate the potential crosstalk between lipid metabolism and immune response [28]. By “immunoediting,” tumor cells interact with the human immune system in the origin and progression of tumors, and infiltrating lymphocytes in the tumor microenvironment have been found to mediate adaptive immunity [29]. Therefore, the lowest infiltration of immune cells may pave the way for the worst clinical outcome of patients in Cluster 1. Patients in the Cluster 2 subtype exhibited more favorable clinical outcomes, whilst patients in the Cluster 1 subtype exhibited the worst overall survival. Functional and signaling pathway enrichment analysis further showed that overlapping DEGs among the three subtypes mainly participated in the regulation of cell cycle and in some cancer-associated pathways, indicating an interface between lipid metabolism and tumor cell uncontrolled growth [4, 5]. Our study, for the first time, stratified the LUAD patients based on LMRG and provided novel insights into predicting the efficacy of patients' survival, as well as potential biomarkers for the response to immunotherapy and targets for immunotherapy.

Given that LMRGs could distinguish patients' clinical and molecular features, we further developed a seven-LMRGs signature that could stratify patients with high or low risk of poor overall survival. Among the seven biomarker genes (CHRDL1, GAPDH, GNPNAT1, HTATIP2, MFI2, PKP2, and RGS20) discovered by the present study, Chordin-like 1 (CHRDL1) is a bone morphogenetic protein (BMP) antagonist. BMPs have emerged as important modulators of cancer aggressiveness. As a tumor suppressor, CHRDL1 is down-expressed in various cancer tissues [30–32], and a high CHRDL1 expression level induced decreased tumor progression [31, 33]. Glyceraldehyde-3-phosphate dehydrogenase (GAPDH) is a pivotal enzyme, and it regulated cellular senescence phenotype in A549 cells via modulating the AMPK network [34], a key pathway implicated in cancer cell lipid metabolism. The relationship between glucosamine 6-phosphate N-acetyltransferase 1 (GNPNAT1/GNA1) and cancer was seldom reported. GNPNAT1 is a key enzyme in the biosynthesis of uridine diphosphate-N-acetylglucosamine, which is an important donor substrate for N-linked glycosylation and thus participates in the regulation of cell growth [35]. HIV-1 Tat interactive protein 2 (HTATIP2) is also a tumor suppressor; by enhancing the HIF2*α*-regulated *β*-catenin/c-Myc/MCL-1 signaling, HTATIP2's deletion increases tumor metabolic plasticity, that is, enable tumor cells to exploit alternative metabolic pathways for replenishing TCA cycle intermediates, to avoid dependence on carbon sources from glutamine and fatty acid, thus inducing LUAD cell survival and proliferation [36]. Plakophilin 2 (PKP2) encodes a plakophilin protein that belongs to the member of desmosomal proteins. PKP2 has been verified overexpressed in several types of human cancers including lung cancer; it is an unfavorable prognostic biomarker for LUAD patients but not for LUSC patients [37]. In addition, it exhibits oncogenic roles through activating EGFR signaling pathway in LUAD cells [37]. Although melanotransferrin (MELTF, MFI2) [38] and regulator of G protein signaling 20 (RGS20) [39] have been reported as regulators in human malignancies, seldom were known about the correlation of both genes with LUAD. Although some of the previous studies have identified these genes as prognostic markers in LUAD, they were limited by just a single gene detected, small sample sizes, and lack of independent validation. The use of the LASSO Cox regression model [40] and nomogram [41] allowed us to integrate multiple genes into one tool, which has significantly greater prognostic accuracy than that of a single gene alone or even some previous reported gene signatures.

Some limitations of this study should be taken into consideration. Although TCGA and two GEO data sets enrolled both Caucasian and Japanese populations, this present study may not include patients with LUAD from other areas loading distinct genetic phenotypes and clinical characteristics, making it susceptible to the inherent biases of such a study format. Clearly, our results should be further validated by a prospective study in some worldwide multicenter clinical studies. Moreover, its prognostic role in early LUAD must be further evaluated. In addition, despite growing studies that began focusing on the interaction of tumor cells and associated lipid metabolism in human malignancies, most LMRGs are not yet functionally annotated in LUAD, and the biofunctions of our seven genes have not yet been fully investigated in previous studies. Although the biological functions of the predictive genes were annotated using computational methods, additional studies should be performed to further reveal the mechanisms of the genes involved in the tumorigenesis of LUAD. Furthermore, more evidences are required to find out the biological foundation of their dysregulation in LUAD.

Cancer is a complex disease, and the interaction between the tumor and its microenvironment plays an important role in the progression of cancer disease. The current clinical stratification scheme focuses on tumor histopathology and molecular characteristics of tumor cells [42]. With further research, people have found that different immune cells are involved in different stages of tumor progression and strive to explore how to use the immune environment determinants that affect tumor development in treatment. Studies have shown that lipid metabolism alteration may be a potential indicator of tumor malignancy and thus prognosis. Therefore, we established a seven-gene signature in LUAD, trying to connect the immunity and lipid metabolism in series in order to improve the accuracy of prognosis and prediction information. Our research results show that in patients with different levels of immune cell infiltration, the mRNA expression levels of 7 genes were different. Our model suggests that different expression levels indicate different survival risks. The survival risk of the high infiltration group is higher than the low infiltration group. The risk model suggests that we cannot measure the survival risk of patients solely by the infiltration level of certain types of immune cells, which reflects the value of our 7-gene signature model in risk prediction. Biomarkers can represent the status of the tumor microenvironment. For example, the high expression level of PD-1 in some cancer may represent the immunosuppressive status. Biomarkers can be used to assess the immune response status of the tumor microenvironment of different patients and find more suitable treatment options for the patients.

In summary, for the first time, we profiled the lipid metabolism phenotype in LUAD, and our study may provide a better assessment of the LMRG-based classification of LUAD. We uncovered the prognostic value of LMRG in LUAD and identified a lipid metabolism-related signature that could classify LUAD patients with high- and low-risk groups of unfavorable survival. This method might, therefore, help with patient counselling and individualized management of patients with LUAD.

## Figures and Tables

**Table 1:** Clinical and pathologic characteristics of patients in the preprocessed TCGA and GEO LUAD data sets.

Characteristic		TCGA data set (*n* = 490)	Training set (*n* = 441)	GSE31210 data set (*n* = 226)	GSE50081 data set (*n* = 129)
Age (years)	<65	213	192	164	42
	≥65	267	241	62	87

Survival state	Alive	312	178	191	76
	Dead	280	161	35	53

Gender	Female	262	238	121	62
	Male	238	203	105	67

T stage	T1	163	146	—	44
	T2	263	243	—	83
	T3	42	37	—	2
	T4	18	13	—	—

N stage	N0	317	290	—	129
	N1	92	80	—	—
	N2	68	40	—	—

M stage	M0	324	289	—	—
	M1	24	22	—	—
	MX	138	126	—	—

Tumor stage	Stage I	263	242	168	93
	Stage II	115	105	58	36
	Stage III	79	65	—	—
	Stage IV	25	22	—	—

TP53 mutation	Present	253	233	—	—
	Absent	237	237	—	—

KRAS mutation	Present	140	126	—	—
	Absent	350	315	—	—

RP1L1 mutation	Present	97	89	—	—
	Absent	393	352	—	—

Smoking history	1 year	68	62	—	—
	2 years	116	104	—	—
	3 years	126	112	—	—
	4 years	162	156	—	—

**Table 2:** RT-qPCR primer of seven genes.

Genes	Primer
CHRDL1	F 5′- AGAGTGGGTGAGAGATGGCA-3′ R 5′-GGTAAGGAGTCTTCTGGGCA-3′

GAPDH	F 5′- GTCTCCTCTGACTTCAACAGCG-3′ R 5′-ACCACCCTGTTGCTGTAGCCAA-3′

GNPNAT1	F 5′- CACTGGTGGGGGAGAGTC-3′ R 5′- CATTTTTCTAGTAAGGTCCGTAGAG-3′

HTATIP2	F 5′- AGGGAAGGTGGGATGCTCT-3′ R 5′- TGTTTCGGCCATGCTGGG-3′

MFI2	F 5′- GACAACACAAACGGCCACAA-3′ R 5′- TGTGGTCGTCTCCAAACAGG-3′

PKP2	F 5′- CTGAAGCTCGGAAGAGGGTTA-3′ R 5′- GCCATTCCTACTTCTTAAATTGACT-3′

RGS20	F 5′- CTTCCCACGAACTCAGAGCAGA-3′ R 5′- TCCTTCCTGCTGGAGTGACCAT-3′

*β*-actin	F 5′-AGTCATTCCAAATATGAGATGCGTT-3′ R 5′- TGCTATCACCTCCCCTGTGT-3′

**Table 3:** Univariate Cox regression analysis result of seven genes in the training set.

Symbol	Coefficient	Hazard ratio	Z-score	*P*-value	Low 95% CI	High 95% CI
CHRDL1	−0.010	0.9898	−2.144	0.03200	0.9805	0.9991
GAPDH	0.000	1.0001	1.812	0.07000	1.0000	1.0002
GNPNAT1	0.011	1.0106	4.417	0.00001	1.0059	1.0153
HTATIP2	0.004	1.0039	2.445	0.01450	1.0008	1.0070
MFI2	0.006	1.0064	1.896	0.05800	0.9998	1.0132
PKP2	0.008	1.0085	2.016	0.04380	1.0002	1.0168
RGS20	0.028	1.0288	1.472	0.14110	0.9906	1.0683

## Data Availability

The data sets generated and analyzed during the current study are available in the TCGA repository (https://portal.gdc.cancer.gov/) and the GEO repository (https://www.ncbi.nlm.nih.gov/geo/).

## Supplementary Materials

Supplementary Materials Supplementary Figure 1: (A) NMF rank survey of cophenetic, RSS, and dispersion; (B) distribution of clinicopathological parameters in the three subtypes; (C) proportions of B cell, CD4^+^T cell, CD8^+^T cell, neutrophil, macrophage, and dendritic cell (DC) in the three subtypes; and (D) distribution of the tumor immune estimation resource, tumor stromal, immune, and estimate scores in the three subtypes. Supplementary Figure 2: (A) Hierarchical clustering for identification of samples with outliers; (B and C) analysis of network topology for various soft-thresholding powers; (D) the Venn diagrams of the overlapping genes among the DEGs in each molecular subtype; (E) enriched top 20 KEGG pathways of overlapping genes; and (F–H) enriched top 20 gene oncology (GO) cellular component, molecular function, and biological process of overlapping genes. The color from red to blue represents the significance of *P* value; the redder color represents smaller *P* value; the dot size represents the number of genes enriched into the pathway; larger number represents a larger value. Supplementary Figure 3: (A) Confidence intervals of each lambda; (B) trajectory change of each independent variable, the *X*-axis represents the log value of the independent variable lambda and the *Y*-axis represents the coefficient of the independent variable; (C-I) Kaplan–Meier survival analysis of overall survival for patients with high or low expression of CHRDL1 (C), GAPDH (D), GNPNAT1 (E), HTATIP2 (F), MFI2 (G), PKP2 (H), and RGS20 (I) in the training set. Supplementary Figure 4. Forest plot of the univariate (A) and multivariate (B) Cox regression analyses in the TCGA LUAD data set. Supplementary Figure 5. Kaplan–Meier and ROC curve survival analysis of the gene signature of Shukla et al. (A), Li et al. (B), Yue et al. (C), and Liu et al. (D).
