# Supplementary material for: A Lipid Metabolism-Based Seven-Gene Signature Correlates with the Clinical Outcome of Lung Adenocarcinoma
Source: J Oncol. 2022 Feb 11;2022:9913206. doi: 10.1155/2022/9913206 (PMC8856807; doi:10.1155/2022/9913206)
Supplement: Supplementary Materials — Supplementary Figure 1: (A) NMF rank survey of cophenetic, RSS, and dispersion; (B) distribution of clinicopathological parameters in the three subtypes; (C) proportions of B cell, CD4+T cell, CD8+T cell, neutrophil, macrophage, and dendritic cell (DC) in the three subtypes; and (D) distribution of the tumor immune estimation resource, tumor stromal, immune, and estimate scores in the three subtypes. Supplementary Figure 2: (A) Hierarchical clustering for identification of samples with outliers; (B and C) analysis of network topology for various soft-thresholding powers; (D) the Venn diagrams of the overlapping genes among the DEGs in each molecular subtype; (E) enriched top 20 KEGG pathways of overlapping genes; and (F–H) enriched top 20 gene oncology (GO) cellular component, molecular function, and biological process of overlapping genes. The color from red to blue represents the significance of P value; the redder color represents smaller P value; the dot size represents the number of genes enriched into the pathway; larger number represents a larger value. Supplementary Figure 3: (A) Confidence intervals of each lambda; (B) trajectory change of each independent variable, the X-axis represents the log value of the independent variable lambda and the Y-axis represents the coefficient of the independent variable; (C-I) Kaplan–Meier survival analysis of overall survival for patients with high or low expression of CHRDL1 (C), GAPDH (D), GNPNAT1 (E), HTATIP2 (F), MFI2 (G), PKP2 (H), and RGS20 (I) in the training set. Supplementary Figure 4. Forest plot of the univariate (A) and multivariate (B) Cox regression analyses in the TCGA LUAD data set. Supplementary Figure 5. Kaplan–Meier and ROC curve survival analysis of the gene signature of Shukla et al. (A), Li et al. (B), Yue et al. (C), and Liu et al. (D). [file 9913206.f1.zip › 9913206.f1/Supplementary Table 2.docx]

| **Supplementary Table 2** Immune score of each sample in TCGA dataset | | | |
| --- | --- | --- | --- |
| Sample | StromalScore | ImmuneScore | ESTIMATEScore |
| TCGA-44-6778 | 5786.818 | 9597.072 | 15383.891 |
| TCGA-69-8254 | 3663.846 | 7398.534 | 11062.379 |
| TCGA-55-6970 | 4694.336 | 7590.726 | 12285.062 |
| TCGA-49-6743 | 5327.801 | 6701.461 | 12029.262 |
| TCGA-05-5420 | 5637.161 | 9421.63 | 15058.791 |
| TCGA-49-4506 | 4079.909 | 7592.045 | 11671.955 |
| TCGA-49-AARE | 3868.59 | 6238.037 | 10106.627 |
| TCGA-L9-A443 | 4647.477 | 6208.402 | 10855.879 |
| TCGA-73-4676 | 4223.283 | 6225.89 | 10449.173 |
| TCGA-78-7148 | 3858.162 | 6244.031 | 10102.193 |
| TCGA-05-4384 | 4452.124 | 6738.228 | 11190.352 |
| TCGA-78-7153 | 3142.786 | 5384.186 | 8526.972 |
| TCGA-55-8204 | 4487.184 | 6853.777 | 11340.961 |
| TCGA-NJ-A55R | 3445.984 | 4804.497 | 8250.48 |
| TCGA-MP-A4TE | 2015.542 | 3811.117 | 5826.658 |
| TCGA-MP-A4T7 | 3270.874 | 6726.912 | 9997.787 |
| TCGA-L9-A5IP | 3147.827 | 6129.189 | 9277.016 |
| TCGA-99-8033 | 4011.915 | 7319.265 | 11331.179 |
| TCGA-97-A4M3 | 4425.759 | 5139.012 | 9564.771 |
| TCGA-97-8176 | 3656.197 | 6259.511 | 9915.709 |
| TCGA-97-7941 | 4591.077 | 6699.299 | 11290.376 |
| TCGA-95-7043 | 3170.132 | 4002.012 | 7172.144 |
| TCGA-93-8067 | 3404.12 | 5587.63 | 8991.749 |
| TCGA-91-6830 | 5921.65 | 7555.212 | 13476.862 |
| TCGA-86-A4D0 | 2190.527 | 5020.379 | 7210.906 |
| TCGA-86-8674 | 2460.659 | 3907.078 | 6367.737 |
| TCGA-86-8358 | 3294.208 | 5174.579 | 8468.787 |
| TCGA-86-8073 | 5184.253 | 6352.391 | 11536.644 |
| TCGA-78-8660 | 4304.399 | 8272.292 | 12576.69 |
| TCGA-78-7220 | 3029.247 | 4572.556 | 7601.803 |
| TCGA-78-7167 | 2940.491 | 4321.964 | 7262.455 |
| TCGA-78-7166 | 2429.362 | 5598.285 | 8027.647 |
| TCGA-78-7160 | 5779.167 | 8265.616 | 14044.784 |
| TCGA-78-7154 | 3740.005 | 5301.456 | 9041.461 |
| TCGA-78-7150 | 4435.648 | 4645.549 | 9081.197 |
| TCGA-75-7027 | 5041.434 | 5349.999 | 10391.433 |
| TCGA-73-4670 | 4578.05 | 5594.046 | 10172.096 |
| TCGA-73-4666 | 4488.764 | 8495.515 | 12984.28 |
| TCGA-73-4659 | 5390.571 | 6552.34 | 11942.911 |
| TCGA-69-A59K | 3689.456 | 6790.4 | 10479.856 |
| TCGA-69-8255 | 2551.988 | 7842.117 | 10394.105 |
| TCGA-69-8253 | 3479.096 | 6085.608 | 9564.705 |
| TCGA-67-3771 | 4918.101 | 7366.541 | 12284.642 |
| TCGA-62-A471 | 1566.318 | 4856.968 | 6423.287 |
| TCGA-62-8398 | 4177.939 | 6792.929 | 10970.867 |
| TCGA-55-A4DF | 3486.978 | 7624.601 | 11111.579 |
| TCGA-55-A492 | 2998.215 | 5434.87 | 8433.085 |
| TCGA-55-A48Y | 5578.154 | 6337.248 | 11915.401 |
| TCGA-55-8620 | 2351.881 | 6278.866 | 8630.747 |
| TCGA-55-8505 | 4766.96 | 4817.691 | 9584.651 |
| TCGA-55-8302 | 3692.858 | 6770.079 | 10462.938 |
| TCGA-55-8208 | 6742.413 | 9513.29 | 16255.703 |
| TCGA-55-8085 | 4173.438 | 7811.537 | 11984.974 |
| TCGA-55-7995 | 4360.143 | 8765.556 | 13125.7 |
| TCGA-55-7570 | 2818.869 | 4075.025 | 6893.894 |
| TCGA-55-6984 | 2918.833 | 6145.076 | 9063.908 |
| TCGA-55-6972 | 123.5944 | 2988.829 | 3112.4234 |
| TCGA-55-6968 | 4246.656 | 7377.359 | 11624.015 |
| TCGA-55-5899 | 4992.418 | 6576.53 | 11568.948 |
| TCGA-55-1596 | 3116.059 | 5624.372 | 8740.431 |
| TCGA-53-A4EZ | 2184.571 | 4900.684 | 7085.255 |
| TCGA-50-6592 | 5110.781 | 7926.209 | 13036.99 |
| TCGA-50-5936 | 5026.171 | 6480.383 | 11506.554 |
| TCGA-50-5072 | 4416.222 | 5619.087 | 10035.309 |
| TCGA-50-5051 | 3143.652 | 4860.061 | 8003.713 |
| TCGA-49-AAR9 | 2401.159 | 4158.495 | 6559.654 |
| TCGA-49-6742 | 2574.322 | 4656.274 | 7230.596 |
| TCGA-49-4514 | 3158.823 | 6683.236 | 9842.059 |
| TCGA-49-4486 | 1434.898 | 4434.139 | 5869.037 |
| TCGA-44-A4SS | 5427.906 | 8309.423 | 13737.33 |
| TCGA-44-7670 | 2246.708 | 4728.028 | 6974.735 |
| TCGA-44-7669 | 4233.554 | 6894.379 | 11127.933 |
| TCGA-44-7660 | 2790.002 | 5615.63 | 8405.632 |
| TCGA-44-6145 | 6025.19 | 8705.755 | 14730.945 |
| TCGA-44-2655 | 4157.986 | 6978.781 | 11136.767 |
| TCGA-38-4631 | 3221.335 | 5972.914 | 9194.248 |
| TCGA-35-5375 | 4373.211 | 7061.611 | 11434.822 |
| TCGA-05-5429 | 3927.411 | 5367.781 | 9295.192 |
| TCGA-05-5428 | 4760.591 | 6552.775 | 11313.366 |
| TCGA-05-4422 | 3088.899 | 7900.376 | 10989.276 |
| TCGA-05-4389 | 3983.828 | 7998.043 | 11981.871 |
| TCGA-05-4418 | 4542.671 | 7648.638 | 12191.309 |
| TCGA-73-7499 | 4540.327 | 8069.804 | 12610.132 |
| TCGA-49-4512 | 5742.726 | 6694.504 | 12437.231 |
| TCGA-78-7163 | 530.1481 | 4542.725 | 5072.8731 |
| TCGA-69-7980 | 4796.504 | 7390.282 | 12186.786 |
| TCGA-62-8394 | 3766.945 | 6049.886 | 9816.831 |
| TCGA-49-AAR2 | 2604.225 | 6564.594 | 9168.819 |
| TCGA-99-7458 | 6035.665 | 8237.462 | 14273.127 |
| TCGA-97-7554 | 6451.295 | 7005.51 | 13456.805 |
| TCGA-97-8177 | 6308.454 | 8182.997 | 14491.451 |
| TCGA-64-5779 | 4941.437 | 6817.488 | 11758.924 |
| TCGA-55-A491 | 4728.512 | 7321.706 | 12050.218 |
| TCGA-05-4405 | 6001.139 | 6678.398 | 12679.538 |
| TCGA-49-4490 | 4621.553 | 6028.32 | 10649.873 |
| TCGA-05-5715 | 5466.344 | 6781.853 | 12248.198 |
| TCGA-78-7149 | 3911.123 | 4967.16 | 8878.282 |
| TCGA-95-A4VP | 4719.95 | 7318.134 | 12038.084 |
| TCGA-93-A4JO | 5066.702 | 8653.106 | 13719.808 |
| TCGA-55-7907 | 4985.17 | 7461.206 | 12446.376 |
| TCGA-75-5147 | 4886.878 | 7465.564 | 12352.442 |
| TCGA-S2-AA1A | 5852.52 | 8798.761 | 14651.281 |
| TCGA-O1-A52J | 3739.62 | 7434.033 | 11173.653 |
| TCGA-NJ-A4YG | 4804.391 | 7112.511 | 11916.902 |
| TCGA-MP-A5C7 | 2036.223 | 3982.643 | 6018.867 |
| TCGA-MP-A4TJ | 6172.587 | 9646.216 | 15818.803 |
| TCGA-MP-A4TH | 4756.378 | 8435.625 | 13192.004 |
| TCGA-MP-A4TD | 5761.504 | 6887.08 | 12648.584 |
| TCGA-MP-A4T9 | 5227.753 | 7369.541 | 12597.294 |
| TCGA-MP-A4T6 | 1859.09 | 6663.379 | 8522.469 |
| TCGA-L9-A7SV | 2063.738 | 4459.371 | 6523.109 |
| TCGA-L9-A50W | 3589.087 | 6389.761 | 9978.848 |
| TCGA-L9-A444 | 5494.24 | 9146.331 | 14640.571 |
| TCGA-L4-A4E6 | 6146.209 | 9149.511 | 15295.72 |
| TCGA-J2-A4AG | 5367.051 | 7922.178 | 13289.229 |
| TCGA-J2-A4AE | 3238.955 | 7235.741 | 10474.695 |
| TCGA-99-AA5R | 6134.597 | 9308.208 | 15442.805 |
| TCGA-97-A4M7 | 5163.019 | 8369.692 | 13532.711 |
| TCGA-97-A4M6 | 5211.374 | 8338.273 | 13549.647 |
| TCGA-97-A4M5 | 5426.689 | 7508.783 | 12935.471 |
| TCGA-97-A4M2 | 4906.859 | 8845.262 | 13752.121 |
| TCGA-97-A4M1 | 4353.351 | 7243.256 | 11596.607 |
| TCGA-97-A4M0 | 3711.503 | 8046.763 | 11758.266 |
| TCGA-97-A4LX | 6419.777 | 9266.265 | 15686.041 |
| TCGA-97-8552 | 4328.113 | 7685.649 | 12013.761 |
| TCGA-97-8179 | 4246.385 | 5629.807 | 9876.192 |
| TCGA-97-8174 | 5693.979 | 7418.056 | 13112.035 |
| TCGA-97-8172 | 6056.486 | 8358.593 | 14415.079 |
| TCGA-97-8171 | 1856.263 | 3933.87 | 5790.133 |
| TCGA-97-7553 | 6102.759 | 9438.28 | 15541.039 |
| TCGA-97-7552 | 5373.648 | 9323.386 | 14697.034 |
| TCGA-97-7547 | 4751.075 | 6478.631 | 11229.706 |
| TCGA-97-7546 | 5902.164 | 7709.435 | 13611.599 |
| TCGA-95-8039 | 4604.032 | 7900.627 | 12504.659 |
| TCGA-95-7948 | 3311.991 | 5195.545 | 8507.536 |
| TCGA-93-A4JP | 5053.313 | 7238.569 | 12291.882 |
| TCGA-93-A4JN | 4775.026 | 6721.517 | 11496.543 |
| TCGA-93-7348 | 5443.901 | 6742.51 | 12186.412 |
| TCGA-93-7347 | 6225.142 | 9018.054 | 15243.196 |
| TCGA-91-A4BD | 2913.201 | 7696.793 | 10609.995 |
| TCGA-91-8497 | 5413.28 | 8194.748 | 13608.029 |
| TCGA-91-8496 | 4600.799 | 8198.854 | 12799.653 |
| TCGA-91-7771 | 5799.387 | 8392.934 | 14192.321 |
| TCGA-91-6849 | 4655.236 | 7645.23 | 12300.465 |
| TCGA-91-6835 | 6187.459 | 9470.781 | 15658.24 |
| TCGA-91-6828 | 5536.567 | 8226.191 | 13762.759 |
| TCGA-86-A4P8 | 6107.902 | 9623.485 | 15731.388 |
| TCGA-86-A4P7 | 5462.006 | 8132.761 | 13594.767 |
| TCGA-86-A456 | 4773.792 | 7511.708 | 12285.499 |
| TCGA-86-8671 | 7005.685 | 9980.222 | 16985.907 |
| TCGA-86-8669 | 3897.201 | 6903.135 | 10800.336 |
| TCGA-86-8668 | 5851.99 | 7333.087 | 13185.077 |
| TCGA-86-8280 | 5813.58 | 8260.636 | 14074.216 |
| TCGA-86-8056 | 4448.572 | 6925.791 | 11374.362 |
| TCGA-86-7714 | 4513.03 | 6695.64 | 11208.67 |
| TCGA-86-7713 | 2461.766 | 4343.977 | 6805.743 |
| TCGA-86-6851 | 4925.257 | 9265.256 | 14190.513 |
| TCGA-78-8648 | 7984.891 | 9520.291 | 17505.182 |
| TCGA-78-7633 | 3343.674 | 4558.91 | 7902.584 |
| TCGA-78-7540 | 2922.166 | 5961.12 | 8883.285 |
| TCGA-78-7539 | 4063.009 | 8077.214 | 12140.223 |
| TCGA-78-7537 | 4487.028 | 5785.605 | 10272.633 |
| TCGA-78-7162 | 5153.724 | 7445.595 | 12599.319 |
| TCGA-78-7158 | 2887.428 | 5157.497 | 8044.925 |
| TCGA-78-7156 | 2686.096 | 4749.416 | 7435.512 |
| TCGA-78-7152 | 4331.433 | 7193.089 | 11524.523 |
| TCGA-78-7147 | 2456.876 | 5975.119 | 8431.995 |
| TCGA-78-7143 | 2872.021 | 6638.585 | 9510.606 |
| TCGA-75-7025 | 5184.974 | 7547.154 | 12732.128 |
| TCGA-75-6212 | 4720.747 | 7951.796 | 12672.543 |
| TCGA-75-6206 | 4892.57 | 6322.381 | 11214.951 |
| TCGA-75-5146 | 4398.518 | 7247.898 | 11646.416 |
| TCGA-73-7498 | 4938.506 | 6658.755 | 11597.26 |
| TCGA-73-4677 | 3980.19 | 6553.352 | 10533.542 |
| TCGA-73-4662 | 4833.015 | 7802.898 | 12635.913 |
| TCGA-69-8453 | 5328.054 | 8982.256 | 14310.31 |
| TCGA-69-7764 | 3711.335 | 5961.791 | 9673.127 |
| TCGA-69-7763 | 5785.618 | 6488.6 | 12274.218 |
| TCGA-69-7761 | 5457.665 | 8704.509 | 14162.175 |
| TCGA-67-6217 | 4641.518 | 7649.866 | 12291.384 |
| TCGA-67-4679 | 5233.1 | 7390.293 | 12623.393 |
| TCGA-67-3774 | 4775.75 | 7585.577 | 12361.327 |
| TCGA-67-3773 | 5450.697 | 8047.015 | 13497.712 |
| TCGA-67-3772 | 6158.144 | 7426.421 | 13584.565 |
| TCGA-67-3770 | 4284.027 | 7425.702 | 11709.729 |
| TCGA-64-5778 | 3491.917 | 8386.52 | 11878.437 |
| TCGA-64-1681 | 4820.879 | 6990.95 | 11811.828 |
| TCGA-64-1680 | 2774.672 | 5488.809 | 8263.481 |
| TCGA-62-A470 | 3119.927 | 5408.133 | 8528.06 |
| TCGA-62-A46Y | 3716.808 | 7929.409 | 11646.217 |
| TCGA-62-A46V | 4539.338 | 5419.558 | 9958.897 |
| TCGA-62-A46S | 3756.373 | 6575.999 | 10332.372 |
| TCGA-62-A46R | 4413.68 | 7509.703 | 11923.384 |
| TCGA-62-A46P | 3208.139 | 5237.328 | 8445.467 |
| TCGA-62-8397 | 4000.085 | 6707.279 | 10707.364 |
| TCGA-62-8395 | 5022.951 | 5722.097 | 10745.048 |
| TCGA-55-A57B | 4993.872 | 7054.829 | 12048.701 |
| TCGA-55-A4DG | 3359.663 | 6850.409 | 10210.073 |
| TCGA-55-A48X | 4701.27 | 8062.623 | 12763.893 |
| TCGA-55-8621 | 6373.264 | 8972.743 | 15346.007 |
| TCGA-55-8619 | 5678.652 | 9062.379 | 14741.031 |
| TCGA-55-8616 | 3674.302 | 5854.14 | 9528.442 |
| TCGA-55-8514 | 3476.573 | 6217.605 | 9694.178 |
| TCGA-55-8513 | 5293.337 | 8270.985 | 13564.322 |
| TCGA-55-8512 | 3133.93 | 5656.251 | 8790.182 |
| TCGA-55-8510 | 5668.449 | 8502.583 | 14171.032 |
| TCGA-55-8207 | 6263.524 | 7498.89 | 13762.414 |
| TCGA-55-8206 | 5218.953 | 8440.428 | 13659.381 |
| TCGA-55-8097 | 4414.667 | 6589.102 | 11003.769 |
| TCGA-55-8087 | 3753.331 | 5678.064 | 9431.396 |
| TCGA-55-7914 | 4256.875 | 6748.711 | 11005.586 |
| TCGA-55-7816 | 7174.186 | 8227.319 | 15401.505 |
| TCGA-55-7728 | 3767.465 | 9110.225 | 12877.69 |
| TCGA-55-7725 | 3832.102 | 8026.903 | 11859.006 |
| TCGA-55-7724 | 5890.53 | 7998.278 | 13888.808 |
| TCGA-55-7573 | 4902.368 | 7688.012 | 12590.38 |
| TCGA-55-7284 | 5155.442 | 7513.116 | 12668.559 |
| TCGA-55-7283 | 4092.84 | 6856.105 | 10948.945 |
| TCGA-55-7227 | 5922.957 | 8071.991 | 13994.949 |
| TCGA-55-6986 | 3968.678 | 6807.215 | 10775.893 |
| TCGA-55-6983 | 5383.087 | 7828.428 | 13211.514 |
| TCGA-55-1592 | 4395.111 | 6518.618 | 10913.73 |
| TCGA-53-7626 | 5925.477 | 8891.38 | 14816.857 |
| TCGA-50-8460 | 4844.604 | 7917.752 | 12762.356 |
| TCGA-50-8457 | 5840.727 | 8393.742 | 14234.468 |
| TCGA-50-6597 | 3585.333 | 7175.663 | 10760.996 |
| TCGA-50-5946 | 2022.168 | 3895.811 | 5917.979 |
| TCGA-50-5944 | 5746.521 | 6171.766 | 11918.287 |
| TCGA-50-5942 | 4560.27 | 6319.986 | 10880.256 |
| TCGA-50-5935 | 4655.145 | 7097.934 | 11753.078 |
| TCGA-50-5932 | 3389.725 | 5213.182 | 8602.907 |
| TCGA-50-5055 | 6911.761 | 9477.846 | 16389.607 |
| TCGA-50-5049 | 7465.874 | 9772.194 | 17238.068 |
| TCGA-49-AARR | 5133.22 | 7686.942 | 12820.162 |
| TCGA-49-AARQ | 2012.314 | 6339.622 | 8351.936 |
| TCGA-49-AARN | 3936.441 | 6410.61 | 10347.051 |
| TCGA-49-AAR0 | 4156.722 | 7538.355 | 11695.077 |
| TCGA-49-6744 | 6691.699 | 8764.799 | 15456.498 |
| TCGA-49-4510 | 3687.259 | 5542.345 | 9229.604 |
| TCGA-49-4501 | 5442.333 | 7496.522 | 12938.855 |
| TCGA-44-A4SU | 3920.469 | 6314.308 | 10234.777 |
| TCGA-44-A47A | 4070.389 | 7603.774 | 11674.163 |
| TCGA-44-8120 | 5348.701 | 6472.044 | 11820.744 |
| TCGA-44-7671 | 4790.833 | 4896.775 | 9687.608 |
| TCGA-44-7659 | 3289.012 | 6324.547 | 9613.559 |
| TCGA-44-6776 | 3061.996 | 4773.15 | 7835.145 |
| TCGA-44-6775 | 5871.7 | 6455.398 | 12327.099 |
| TCGA-44-6148 | 5085.267 | 6598.524 | 11683.791 |
| TCGA-44-6147 | 5525.111 | 7273.749 | 12798.86 |
| TCGA-44-6146 | 4177.292 | 4247.583 | 8424.875 |
| TCGA-44-5645 | 4673.637 | 6429.028 | 11102.666 |
| TCGA-44-3919 | 5483.183 | 8280.898 | 13764.081 |
| TCGA-44-2666 | 3073.546 | 4823.669 | 7897.215 |
| TCGA-44-2661 | 6351.977 | 9447.491 | 15799.468 |
| TCGA-44-2659 | 5813.576 | 7772.9 | 13586.476 |
| TCGA-44-2657 | 5864.717 | 9309.091 | 15173.808 |
| TCGA-38-A44F | 5398.168 | 8607.996 | 14006.165 |
| TCGA-38-7271 | 6814.234 | 9647.161 | 16461.396 |
| TCGA-38-4626 | 6432.273 | 8456.014 | 14888.287 |
| TCGA-05-5423 | 4718.471 | 8257.858 | 12976.329 |
| TCGA-05-4433 | 4108.375 | 8042.745 | 12151.119 |
| TCGA-05-4424 | 5668.686 | 7443.338 | 13112.024 |
| TCGA-05-4249 | 4770.688 | 7143.077 | 11913.765 |
| TCGA-05-4403 | 4943.834 | 7579.96 | 12523.795 |
| TCGA-55-A494 | 1298.52 | 2527.118 | 3825.638 |
| TCGA-44-A479 | 6048.428 | 9040.006 | 15088.434 |
| TCGA-50-6593 | 5919.334 | 7099.319 | 13018.653 |
| TCGA-55-6971 | 5708.635 | 9202.832 | 14911.467 |
| TCGA-55-8301 | 5859.256 | 9017.679 | 14876.935 |
| TCGA-55-7727 | 3801.765 | 7205.431 | 11007.196 |
| TCGA-05-4434 | 5784.671 | 8601.751 | 14386.421 |
| TCGA-NJ-A7XG | 804.1602 | 4256.5002 | 5060.6604 |
| TCGA-NJ-A4YQ | 4289.846 | 8749.546 | 13039.393 |
| TCGA-NJ-A4YP | 5811.21 | 6558.946 | 12370.155 |
| TCGA-NJ-A4YF | 2612.269 | 4887.068 | 7499.336 |
| TCGA-MP-A4TK | 6707.318 | 7980.409 | 14687.727 |
| TCGA-MP-A4TI | 6638.143 | 9650.414 | 16288.558 |
| TCGA-MP-A4TF | 2078.918 | 5653.73 | 7732.648 |
| TCGA-MP-A4TC | 6123.693 | 7397.305 | 13520.998 |
| TCGA-MP-A4TA | 3589.797 | 6424.912 | 10014.709 |
| TCGA-MP-A4T8 | 4246.443 | 4718.417 | 8964.86 |
| TCGA-MP-A4T4 | 5878.82 | 8747.631 | 14626.451 |
| TCGA-MP-A4SY | 5305.506 | 5916.623 | 11222.129 |
| TCGA-MP-A4SW | 4334.291 | 7566.226 | 11900.517 |
| TCGA-MP-A4SV | 4460.182 | 7557.556 | 12017.738 |
| TCGA-MN-A4N5 | 3129.094 | 6938.279 | 10067.373 |
| TCGA-MN-A4N4 | 5307.421 | 6077.109 | 11384.53 |
| TCGA-MN-A4N1 | 2833.85 | 4384.94 | 7218.79 |
| TCGA-L9-A8F4 | 4380.325 | 8043.784 | 12424.109 |
| TCGA-L9-A743 | 5675.353 | 8829.004 | 14504.356 |
| TCGA-L4-A4E5 | 3032.712 | 5169.371 | 8202.083 |
| TCGA-J2-A4AD | 3380.161 | 4223.963 | 7604.123 |
| TCGA-J2-8194 | 4856.672 | 6569.295 | 11425.967 |
| TCGA-J2-8192 | 7224.295 | 8080.416 | 15304.711 |
| TCGA-99-8032 | 5377.586 | 6175.841 | 11553.427 |
| TCGA-99-8028 | 6870.573 | 9719.31 | 16589.883 |
| TCGA-99-8025 | 4325.446 | 5390.047 | 9715.493 |
| TCGA-97-8547 | 5928.281 | 6579.217 | 12507.498 |
| TCGA-97-8175 | 3895.689 | 7319.973 | 11215.662 |
| TCGA-97-7937 | 3781.272 | 4890.782 | 8672.054 |
| TCGA-95-A4VN | 4824.207 | 8438.917 | 13263.124 |
| TCGA-95-A4VK | 3420.117 | 6752.133 | 10172.25 |
| TCGA-95-8494 | 3396.809 | 6607.479 | 10004.287 |
| TCGA-95-7947 | 3010.113 | 6584.517 | 9594.63 |
| TCGA-95-7944 | 4576.524 | 8437.004 | 13013.528 |
| TCGA-95-7567 | 4724.607 | 6708.138 | 11432.745 |
| TCGA-95-7562 | 4485.399 | 6595.752 | 11081.151 |
| TCGA-95-7039 | 4510.742 | 5938.376 | 10449.118 |
| TCGA-93-A4JQ | 6060.97 | 8538.402 | 14599.373 |
| TCGA-91-A4BC | 6056.811 | 8964.038 | 15020.85 |
| TCGA-91-8499 | 3624.531 | 6366.403 | 9990.934 |
| TCGA-91-6848 | 6339.5 | 8209.758 | 14549.258 |
| TCGA-91-6847 | 538.4039 | 3149.5534 | 3687.9573 |
| TCGA-91-6840 | 3784.463 | 6666.976 | 10451.439 |
| TCGA-91-6836 | 2764.503 | 5406.455 | 8170.958 |
| TCGA-91-6831 | 5503.502 | 6671.675 | 12175.177 |
| TCGA-91-6829 | 6475.149 | 5892.854 | 12368.002 |
| TCGA-86-A4JF | 3967.16 | 7322.95 | 11290.11 |
| TCGA-86-8673 | 4269.76 | 6205.186 | 10474.946 |
| TCGA-86-8585 | 4220.086 | 8170.802 | 12390.888 |
| TCGA-86-8359 | 4152.789 | 7478.047 | 11630.836 |
| TCGA-86-8279 | 4778.789 | 5212.115 | 9990.904 |
| TCGA-86-8278 | 5228.558 | 6751.577 | 11980.135 |
| TCGA-86-8076 | 4952.126 | 8642.48 | 13594.606 |
| TCGA-86-8075 | 6617.507 | 6923.946 | 13541.453 |
| TCGA-86-8055 | 6758.089 | 7132.365 | 13890.453 |
| TCGA-86-8054 | 3370.704 | 4366.39 | 7737.094 |
| TCGA-86-7955 | 581.3324 | 3095.8181 | 3677.1505 |
| TCGA-86-7954 | 5206.148 | 8416.245 | 13622.393 |
| TCGA-86-7953 | 4702.842 | 7745.186 | 12448.028 |
| TCGA-86-7711 | 5676.994 | 7427.586 | 13104.58 |
| TCGA-86-7701 | 5642.931 | 8263.327 | 13906.258 |
| TCGA-86-6562 | 5953.95 | 6406.963 | 12360.913 |
| TCGA-83-5908 | 5056.973 | 8697.213 | 13754.186 |
| TCGA-80-5611 | 3587.013 | 7840.783 | 11427.796 |
| TCGA-80-5608 | 3068.913 | 6055.664 | 9124.577 |
| TCGA-78-8662 | 2469.529 | 4134.874 | 6604.403 |
| TCGA-78-8655 | 4707.976 | 7606.321 | 12314.296 |
| TCGA-78-8640 | 2805.495 | 6379.978 | 9185.473 |
| TCGA-78-7542 | 1881.436 | 5914.227 | 7795.663 |
| TCGA-78-7536 | 3343.198 | 6448.907 | 9792.106 |
| TCGA-78-7535 | 3967.048 | 6982.183 | 10949.231 |
| TCGA-78-7161 | 3487.652 | 4141.083 | 7628.736 |
| TCGA-78-7159 | 4474.719 | 5873.403 | 10348.122 |
| TCGA-78-7155 | 1972.666 | 4053.438 | 6026.104 |
| TCGA-78-7146 | 3174.297 | 5699.612 | 8873.91 |
| TCGA-78-7145 | 4778.206 | 6063.09 | 10841.296 |
| TCGA-75-6214 | 2384.155 | 4729.55 | 7113.704 |
| TCGA-75-5125 | 5013.264 | 8049.861 | 13063.126 |
| TCGA-73-A9RS | 2169.529 | 4977.288 | 7146.817 |
| TCGA-73-4675 | 4773.114 | 5883.592 | 10656.707 |
| TCGA-73-4668 | 4600.561 | 5592.687 | 10193.248 |
| TCGA-73-4658 | 7007.757 | 8568.689 | 15576.446 |
| TCGA-71-8520 | 5169.084 | 5787.949 | 10957.033 |
| TCGA-71-6725 | 2577.793 | 4966.991 | 7544.784 |
| TCGA-69-7979 | 3998.004 | 4838.577 | 8836.582 |
| TCGA-69-7978 | 6261.808 | 9123.858 | 15385.666 |
| TCGA-69-7974 | 5352.887 | 8125.531 | 13478.417 |
| TCGA-69-7973 | 4165.607 | 5291.34 | 9456.947 |
| TCGA-69-7765 | 6567.666 | 7406.628 | 13974.293 |
| TCGA-69-7760 | 3010.989 | 3982.548 | 6993.537 |
| TCGA-67-6216 | 3889.803 | 7616.903 | 11506.706 |
| TCGA-67-6215 | 2933.566 | 6376.876 | 9310.442 |
| TCGA-64-5815 | 7194.873 | 7881.778 | 15076.651 |
| TCGA-64-5781 | 4938.723 | 7566.88 | 12505.603 |
| TCGA-64-5775 | 4286.325 | 5888.575 | 10174.9 |
| TCGA-64-5774 | 3168.509 | 4459.9 | 7628.408 |
| TCGA-64-1679 | 6451.806 | 6738.588 | 13190.394 |
| TCGA-64-1678 | 2341.366 | 4039.053 | 6380.419 |
| TCGA-64-1677 | 2936.72 | 6928.094 | 9864.814 |
| TCGA-64-1676 | 5489.381 | 7932.797 | 13422.178 |
| TCGA-62-A472 | 3211.942 | 6989.267 | 10201.21 |
| TCGA-62-A46O | 908.5491 | 2546.4498 | 3454.9989 |
| TCGA-62-8402 | 2934.61 | 7717.567 | 10652.177 |
| TCGA-62-8399 | 4448.967 | 5760.573 | 10209.54 |
| TCGA-55-A490 | 4943.958 | 6453.953 | 11397.911 |
| TCGA-55-A48Z | 5427.591 | 6788.178 | 12215.768 |
| TCGA-55-8615 | 2875.035 | 4622.965 | 7498 |
| TCGA-55-8614 | 4467.001 | 5945.374 | 10412.375 |
| TCGA-55-8511 | 5536.317 | 7854.181 | 13390.498 |
| TCGA-55-8508 | 4317.344 | 6641.687 | 10959.031 |
| TCGA-55-8507 | 3899.447 | 5787.907 | 9687.354 |
| TCGA-55-8299 | 6948.202 | 8966.754 | 15914.956 |
| TCGA-55-8205 | 5761.941 | 9156.106 | 14918.047 |
| TCGA-55-8203 | 4578.084 | 7296.277 | 11874.361 |
| TCGA-55-8096 | 6029.474 | 7333.813 | 13363.287 |
| TCGA-55-8094 | 343.7257 | 2925.8325 | 3269.5582 |
| TCGA-55-8092 | 4795.731 | 7965.893 | 12761.624 |
| TCGA-55-8091 | 6310.896 | 7844.119 | 14155.015 |
| TCGA-55-8090 | 4502.058 | 6681.694 | 11183.751 |
| TCGA-55-8089 | 5543.72 | 9547.253 | 15090.973 |
| TCGA-55-7994 | 3657.52 | 8312.732 | 11970.252 |
| TCGA-55-7913 | 1539.342 | 4733.951 | 6273.293 |
| TCGA-55-7911 | 3980.668 | 8234.799 | 12215.466 |
| TCGA-55-7910 | 3467.941 | 5325.679 | 8793.621 |
| TCGA-55-7903 | 3324.383 | 6910.524 | 10234.907 |
| TCGA-55-7815 | 6280.797 | 7477.627 | 13758.424 |
| TCGA-55-7726 | 6416.473 | 6387.383 | 12803.856 |
| TCGA-55-7576 | 5043.483 | 6945.517 | 11989 |
| TCGA-55-7574 | 6454.674 | 8838.96 | 15293.634 |
| TCGA-55-7281 | 5773.964 | 8033.082 | 13807.046 |
| TCGA-55-6987 | 5692.155 | 9596.998 | 15289.153 |
| TCGA-55-6985 | 5547.825 | 7893.499 | 13441.324 |
| TCGA-55-6982 | 6089.736 | 7188.303 | 13278.039 |
| TCGA-55-6981 | 4384.251 | 6095.256 | 10479.507 |
| TCGA-55-6980 | 5947.656 | 8082.219 | 14029.875 |
| TCGA-55-6979 | 6327.117 | 9286.813 | 15613.929 |
| TCGA-55-6978 | 7121.845 | 9080.209 | 16202.054 |
| TCGA-55-6975 | 6095.592 | 6058.988 | 12154.58 |
| TCGA-55-6712 | 5281.21 | 8415.444 | 13696.653 |
| TCGA-55-6642 | 6909.709 | 7335.615 | 14245.324 |
| TCGA-55-6543 | 4657.072 | 6983.845 | 11640.917 |
| TCGA-55-1594 | 3522.119 | 6285.34 | 9807.459 |
| TCGA-53-7813 | 2612.413 | 6005.166 | 8617.579 |
| TCGA-53-7624 | 2389.558 | 5471.702 | 7861.26 |
| TCGA-50-8459 | 7527.943 | 8676.573 | 16204.516 |
| TCGA-50-7109 | 4757.07 | 6876.049 | 11633.12 |
| TCGA-50-6595 | 6558.125 | 7461.752 | 14019.876 |
| TCGA-50-6594 | 3954.359 | 5854.249 | 9808.608 |
| TCGA-50-6591 | 1824.887 | 2069.564 | 3894.451 |
| TCGA-50-6590 | 5171.498 | 8383.501 | 13554.999 |
| TCGA-50-5941 | 5778.859 | 9001.722 | 14780.581 |
| TCGA-50-5939 | 5583.188 | 7690.201 | 13273.389 |
| TCGA-50-5933 | 7054.954 | 7777.267 | 14832.221 |
| TCGA-50-5931 | 3044.407 | 3527.747 | 6572.154 |
| TCGA-50-5930 | 5451.926 | 7520.186 | 12972.112 |
| TCGA-50-5068 | 5483.805 | 8608.454 | 14092.259 |
| TCGA-50-5066 | 6181.772 | 8749.646 | 14931.418 |
| TCGA-50-5045 | 7095.226 | 9129.017 | 16224.243 |
| TCGA-50-5044 | 4902.58 | 7133.72 | 12036.3 |
| TCGA-4B-A93V | 2447.971 | 6019.664 | 8467.635 |
| TCGA-49-AARO | 5453.437 | 8319.179 | 13772.615 |
| TCGA-49-AAR4 | 4380.195 | 8512.458 | 12892.653 |
| TCGA-49-AAR3 | 5645.431 | 8743.722 | 14389.153 |
| TCGA-49-AAQV | 3291.909 | 6909.238 | 10201.147 |
| TCGA-49-6767 | 3791.681 | 7222.42 | 11014.101 |
| TCGA-49-6761 | 4304.653 | 7303.658 | 11608.311 |
| TCGA-49-6745 | 6010.85 | 8142.845 | 14153.695 |
| TCGA-49-4507 | 3912.775 | 7722.757 | 11635.532 |
| TCGA-49-4505 | 5470.51 | 8082.332 | 13552.842 |
| TCGA-49-4494 | 3944.587 | 6819.34 | 10763.927 |
| TCGA-49-4488 | 3807.077 | 7072.565 | 10879.642 |
| TCGA-49-4487 | 5707.151 | 8478.629 | 14185.779 |
| TCGA-44-A47G | 5954.553 | 9043.216 | 14997.769 |
| TCGA-44-A47B | 4060.499 | 6809.051 | 10869.549 |
| TCGA-44-8119 | 6139.39 | 6850.692 | 12990.082 |
| TCGA-44-8117 | 4712.208 | 5884.573 | 10596.781 |
| TCGA-44-7672 | 6479.005 | 8869.144 | 15348.149 |
| TCGA-44-7667 | 3288.124 | 4834.536 | 8122.661 |
| TCGA-44-7662 | 6570.384 | 7351.392 | 13921.776 |
| TCGA-44-7661 | 6249.898 | 8319.388 | 14569.286 |
| TCGA-44-6779 | 5832.262 | 8656.498 | 14488.76 |
| TCGA-44-6777 | 7952.787 | 9055.434 | 17008.221 |
| TCGA-44-6774 | 7806.808 | 7228.087 | 15034.895 |
| TCGA-44-5644 | 1843.476 | 3861.949 | 5705.425 |
| TCGA-44-5643 | 2379.596 | 6410.195 | 8789.792 |
| TCGA-44-4112 | 5689.303 | 6555.546 | 12244.849 |
| TCGA-44-3918 | 5696.156 | 7574.782 | 13270.938 |
| TCGA-44-3917 | 3515.775 | 5622.282 | 9138.057 |
| TCGA-44-3398 | 6761.189 | 8352.336 | 15113.525 |
| TCGA-44-3396 | 6581.454 | 8604.424 | 15185.877 |
| TCGA-44-2668 | 5425.177 | 8597.548 | 14022.726 |
| TCGA-44-2665 | 7065.39 | 7450.869 | 14516.26 |
| TCGA-44-2662 | 6035.193 | 6898.403 | 12933.596 |
| TCGA-44-2656 | 5911.474 | 9070.542 | 14982.016 |
| TCGA-38-6178 | 5172.882 | 6107.846 | 11280.728 |
| TCGA-38-4632 | 4818.092 | 7910.588 | 12728.68 |
| TCGA-38-4630 | 3463.491 | 4697.706 | 8161.197 |
| TCGA-38-4629 | 6276.582 | 8434.028 | 14710.61 |
| TCGA-38-4628 | 5169.681 | 6249.667 | 11419.348 |
| TCGA-38-4627 | 8093.822 | 7915.627 | 16009.449 |
| TCGA-38-4625 | 4508.365 | 7288.309 | 11796.674 |
| TCGA-35-4123 | 5789.19 | 8727.371 | 14516.561 |
| TCGA-35-4122 | 5904.501 | 8805.791 | 14710.292 |
| TCGA-05-5425 | 5192.449 | 8544.446 | 13736.895 |
| TCGA-05-4432 | 4335.565 | 7079.017 | 11414.582 |
| TCGA-05-4430 | 6589.78 | 7699.886 | 14289.666 |
| TCGA-05-4427 | 4744.18 | 6877.558 | 11621.738 |
| TCGA-05-4426 | 3506.688 | 6502.317 | 10009.005 |
| TCGA-05-4425 | 5276.009 | 8034.215 | 13310.225 |
| TCGA-05-4420 | 3854.741 | 5026.553 | 8881.294 |
| TCGA-05-4417 | 7017.382 | 8279.67 | 15297.052 |
| TCGA-05-4415 | 2556.095 | 5554.529 | 8110.624 |
| TCGA-05-4402 | 5053.969 | 6890.797 | 11944.766 |
| TCGA-05-4398 | 5190.601 | 7903.792 | 13094.393 |
| TCGA-05-4397 | 3014.049 | 5935.232 | 8949.281 |
| TCGA-05-4396 | 3210.858 | 4892.199 | 8103.057 |
| TCGA-05-4390 | 4734.09 | 5647.358 | 10381.448 |
| TCGA-05-4250 | 5247.344 | 7612.537 | 12859.881 |
| TCGA-05-4382 | 6567.332 | 8074.839 | 14642.17 |
