# Supplementary material for: A Lipid Metabolism-Based Seven-Gene Signature Correlates with the Clinical Outcome of Lung Adenocarcinoma
Source: J Oncol. 2022 Feb 11;2022:9913206. doi: 10.1155/2022/9913206 (PMC8856807; doi:10.1155/2022/9913206)
Supplement: Supplementary Materials — Supplementary Figure 1: (A) NMF rank survey of cophenetic, RSS, and dispersion; (B) distribution of clinicopathological parameters in the three subtypes; (C) proportions of B cell, CD4+T cell, CD8+T cell, neutrophil, macrophage, and dendritic cell (DC) in the three subtypes; and (D) distribution of the tumor immune estimation resource, tumor stromal, immune, and estimate scores in the three subtypes. Supplementary Figure 2: (A) Hierarchical clustering for identification of samples with outliers; (B and C) analysis of network topology for various soft-thresholding powers; (D) the Venn diagrams of the overlapping genes among the DEGs in each molecular subtype; (E) enriched top 20 KEGG pathways of overlapping genes; and (F–H) enriched top 20 gene oncology (GO) cellular component, molecular function, and biological process of overlapping genes. The color from red to blue represents the significance of P value; the redder color represents smaller P value; the dot size represents the number of genes enriched into the pathway; larger number represents a larger value. Supplementary Figure 3: (A) Confidence intervals of each lambda; (B) trajectory change of each independent variable, the X-axis represents the log value of the independent variable lambda and the Y-axis represents the coefficient of the independent variable; (C-I) Kaplan–Meier survival analysis of overall survival for patients with high or low expression of CHRDL1 (C), GAPDH (D), GNPNAT1 (E), HTATIP2 (F), MFI2 (G), PKP2 (H), and RGS20 (I) in the training set. Supplementary Figure 4. Forest plot of the univariate (A) and multivariate (B) Cox regression analyses in the TCGA LUAD data set. Supplementary Figure 5. Kaplan–Meier and ROC curve survival analysis of the gene signature of Shukla et al. (A), Li et al. (B), Yue et al. (C), and Liu et al. (D). [file 9913206.f1.zip › 9913206.f1/Supplementary Table 3 .docx]

| **Supplementary Table 3** List of the 126 prognositic LMRGs in LUAD | | | | | |
| --- | --- | --- | --- | --- | --- |
| GeneSymbol | p.value | HR | Low 95%CI | High 95%CI |  |
| NFYA | 0.660692 | 1.001167 | 0.995965 | 1.006397 |  |
| CREBBP | 0.370144 | 0.994136 | 0.98143 | 1.007006 |  |
| ABCB4 | 0.873229 | 0.992602 | 0.906075 | 1.087392 |  |
| MED24 | 0.703912 | 1.001351 | 0.994404 | 1.008346 |  |
| NR1H4 | 0.05816 | 1.044427 | 0.998499 | 1.092467 |  |
| ALAS1 | 0.405268 | 0.998235 | 0.994092 | 1.002396 |  |
| NR1H3 | 0.650105 | 0.996992 | 0.984099 | 1.010054 |  |
| MED17 | 0.400291 | 1.018618 | 0.97576 | 1.063358 |  |
| THRAP3 | 0.390403 | 1.001794 | 0.997705 | 1.0059 |  |
| MED29 | 0.866298 | 1.000452 | 0.995203 | 1.005729 |  |
| AHRR | 0.786345 | 0.989175 | 0.914314 | 1.070166 |  |
| ME1 | 0.215716 | 1.002299 | 0.998662 | 1.005949 |  |
| NFYC | 0.801089 | 1.001674 | 0.988727 | 1.01479 |  |
| RORA | 0.031638 | 0.932317 | 0.874588 | 0.993856 |  |
| SREBF1 | 0.617514 | 0.998881 | 0.994502 | 1.003279 |  |
| FDFT1 | 0.19904 | 1.001885 | 0.99901 | 1.004769 |  |
| SMARCD3 | 0.046566 | 0.984971 | 0.970391 | 0.99977 |  |
| NCOA1 | 0.646192 | 0.997054 | 0.984572 | 1.009694 |  |
| MED15 | 0.005885 | 1.011909 | 1.00342 | 1.02047 |  |
| EP300 | 0.606141 | 1.002347 | 0.993454 | 1.011321 |  |
| TRIB3 | 0.004724 | 1.005124 | 1.001566 | 1.008693 |  |
| TBL1X | 0.171112 | 1.007153 | 0.996925 | 1.017486 |  |
| MED25 | 0.535406 | 1.004661 | 0.989994 | 1.019546 |  |
| MED26 | 0.070992 | 0.925188 | 0.850298 | 1.006673 |  |
| NRF1 | 0.957813 | 0.999015 | 0.963178 | 1.036185 |  |
| AHR | 0.822496 | 0.999818 | 0.998227 | 1.001411 |  |
| MED13 | 0.300703 | 0.994973 | 0.985523 | 1.004514 |  |
| MED31 | 0.585533 | 1.005775 | 0.985174 | 1.026807 |  |
| PPARGC1A | 0.364803 | 1.010343 | 0.988107 | 1.03308 |  |
| CPT1A | 0.96092 | 0.999804 | 0.991989 | 1.007681 |  |
| APOA5 | 0.320114 | 0.905617 | 0.744846 | 1.101088 |  |
| CCNC | 0.523675 | 0.99725 | 0.988845 | 1.005727 |  |
| MED23 | 0.617641 | 0.9937 | 0.969348 | 1.018665 |  |
| FAM120B | 0.948404 | 0.999302 | 0.97839 | 1.020661 |  |
| HMGCS1 | 0.060033 | 1.003245 | 0.999863 | 1.006639 |  |
| HMGCR | 0.061006 | 1.009727 | 0.999553 | 1.020005 |  |
| FHL2 | 0.015612 | 1.004869 | 1.00092 | 1.008833 |  |
| ACADM | 0.363105 | 1.004313 | 0.995041 | 1.013671 |  |
| APOA1 | 0.22784 | 0.938555 | 0.846643 | 1.040446 |  |
| MED28 | 0.846631 | 0.998035 | 0.978338 | 1.018128 |  |
| NFYB | 0.190257 | 1.014497 | 0.992881 | 1.036583 |  |
| MED13L | 0.168861 | 0.990588 | 0.977333 | 1.004023 |  |
| G0S2 | 0.021522 | 1.000603 | 1.000089 | 1.001117 |  |
| NCOA3 | 0.548173 | 1.00258 | 0.994184 | 1.011047 |  |
| MED20 | 0.452234 | 0.996709 | 0.988178 | 1.005313 |  |
| MED1 | 0.669138 | 1.002291 | 0.991827 | 1.012865 |  |
| NR1D1 | 0.454372 | 1.002983 | 0.995187 | 1.010841 |  |
| SIN3B | 0.130244 | 0.98626 | 0.968743 | 1.004094 |  |
| SLC27A1 | 0.020457 | 0.970681 | 0.946561 | 0.995416 |  |
| HELZ2 | 0.248463 | 1.005921 | 0.995886 | 1.016058 |  |
| MED18 | 0.558728 | 0.995785 | 0.981787 | 1.009983 |  |
| NR1H2 | 0.791993 | 1.000861 | 0.99448 | 1.007283 |  |
| PPARG | 0.004605 | 1.010584 | 1.003251 | 1.017971 |  |
| GPS2 | 0.335374 | 0.969991 | 0.911687 | 1.032023 |  |
| CDK8 | 0.514995 | 1.006788 | 0.986492 | 1.027501 |  |
| MED10 | 0.4578 | 1.001848 | 0.996977 | 1.006742 |  |
| ARNTL | 0.048754 | 0.961824 | 0.925302 | 0.999787 |  |
| MED6 | 0.010967 | 1.032233 | 1.007307 | 1.057776 |  |
| HMGCS2 | 0.046596 | 1.002932 | 1.000044 | 1.005829 |  |
| GRHL1 | 0.424419 | 0.993626 | 0.978156 | 1.00934 |  |
| CLOCK | 0.929828 | 0.999269 | 0.98313 | 1.015673 |  |
| CD36 | 0.495496 | 0.996165 | 0.985221 | 1.007232 |  |
| AGT | 0.928928 | 0.99997 | 0.999317 | 1.000624 |  |
| MED4 | 0.248898 | 0.994241 | 0.984527 | 1.00405 |  |
| TGS1 | 0.497635 | 1.003553 | 0.993319 | 1.013893 |  |
| GLIPR1 | 0.509996 | 0.99584 | 0.983566 | 1.008267 |  |
| NCOA2 | 0.711852 | 0.998011 | 0.98752 | 1.008612 |  |
| CYP1A1 | 0.424259 | 1.001648 | 0.997611 | 1.005702 |  |
| MED9 | 0.974127 | 1.000621 | 0.96377 | 1.038881 |  |
| NCOR1 | 0.961168 | 1.000301 | 0.988266 | 1.012482 |  |
| CARM1 | 0.14062 | 1.008361 | 0.997255 | 1.019591 |  |
| RGL1 | 0.017469 | 0.989361 | 0.980673 | 0.998126 |  |
| ARNT | 0.258756 | 0.994409 | 0.984781 | 1.004132 |  |
| TNFRSF21 | 0.235724 | 1.000664 | 0.999567 | 1.001763 |  |
| TIAM2 | 0.426147 | 1.049567 | 0.931675 | 1.182378 |  |
| PLIN2 | 0.493967 | 1.000833 | 0.998448 | 1.003224 |  |
| MED22 | 0.895068 | 1.001658 | 0.977297 | 1.026627 |  |
| ANKRD1 | 0.680194 | 1.001314 | 0.995083 | 1.007584 |  |
| FADS1 | 0.182977 | 1.005509 | 0.997411 | 1.013673 |  |
| ACSL1 | 0.265211 | 0.998117 | 0.994813 | 1.001432 |  |
| MED21 | 0.055906 | 1.006595 | 0.999834 | 1.013401 |  |
| CDK19 | 0.786255 | 0.996589 | 0.972277 | 1.021508 |  |
| PPARGC1B | 0.578984 | 0.96386 | 0.846344 | 1.097694 |  |
| MED7 | 0.600987 | 1.009822 | 0.973503 | 1.047496 |  |
| MED19 | 0.163725 | 1.011935 | 0.995178 | 1.028974 |  |
| CPT2 | 0.72908 | 0.997383 | 0.982703 | 1.012283 |  |
| APOA2 | 0.404887 | 0.985687 | 0.95281 | 1.019699 |  |
| MED8 | 0.49761 | 0.997414 | 0.98998 | 1.004905 |  |
| MED27 | 0.21836 | 1.005542 | 0.996732 | 1.014431 |  |
| ACOX1 | 0.723208 | 1.001835 | 0.991723 | 1.01205 |  |
| MED11 | 0.84872 | 0.998924 | 0.987937 | 1.010034 |  |
| MED30 | 0.892942 | 1.000788 | 0.989374 | 1.012334 |  |
| ABCA1 | 0.153929 | 0.991901 | 0.980876 | 1.003051 |  |
| PEX11A | 0.745259 | 1.00444 | 0.977951 | 1.031647 |  |
| ANGPTL4 | 6.50E-06 | 1.002378 | 1.001344 | 1.003414 |  |
| CYP7A1 | 0.494202 | 0.329945 | 0.013735 | 7.925859 |  |
| SIN3A | 0.419284 | 1.00815 | 0.988487 | 1.028203 |  |
| NPAS2 | 8.22E-05 | 1.043851 | 1.02179 | 1.066388 |  |
| HDAC3 | 0.667528 | 1.002564 | 0.990917 | 1.014348 |  |
| ARNT2 | 0.856973 | 1.000928 | 0.990884 | 1.011073 |  |
| ESRRA | 0.251681 | 1.003908 | 0.997235 | 1.010626 |  |
| MED16 | 0.752039 | 1.001447 | 0.992505 | 1.01047 |  |
| CHD9 | 0.054737 | 0.971485 | 0.943228 | 1.000588 |  |
| TBL1XR1 | 0.521369 | 1.001641 | 0.996633 | 1.006675 |  |
| MED14 | 0.472201 | 1.004636 | 0.992048 | 1.017384 |  |
| MED12 | 0.742158 | 0.997777 | 0.984633 | 1.011096 |  |
| SP1 | 0.081393 | 1.007283 | 0.999096 | 1.015538 |  |
| RXRA | 0.480264 | 0.995364 | 0.982603 | 1.00829 |  |
| PPARA | 0.408908 | 0.982066 | 0.940779 | 1.025165 |  |
| CYP4A11 | 0.350762 | 0.645886 | 0.257867 | 1.617765 |  |
| MTF1 | 0.21046 | 0.9792 | 0.947512 | 1.011948 |  |
| NCOR2 | 0.884391 | 1.000351 | 0.995635 | 1.005088 |  |
| TXNRD1 | 6.26E-05 | 1.0007 | 1.000357 | 1.001043 |  |
| NCOA6 | 0.507116 | 1.004521 | 0.991221 | 1.017999 |  |
| SREBF2 | 0.307876 | 1.001926 | 0.998227 | 1.005639 |  |
| RXRB | 0.172147 | 0.99321 | 0.983544 | 1.002971 |  |
| CYP51A1 | 0.385043 | 1.019757 | 0.97572 | 1.065783 |  |
| MTMR7 | 0.203985 | 0.988533 | 0.971098 | 1.006281 |  |
| NDUFAB1 | 0.051371 | 1.003602 | 0.999979 | 1.007238 |  |
| ACSM3 | 0.348304 | 0.989816 | 0.968868 | 1.011217 |  |
| BZRAP1 | 0.016386 | 0.957267 | 0.923731 | 0.992022 |  |
| PON1 | 0.702759 | 0.993792 | 0.962509 | 1.026092 |  |
| CROT | 0.826641 | 0.996816 | 0.968766 | 1.025677 |  |
| GDE1 | 0.522866 | 0.998959 | 0.995773 | 1.002155 |  |
| OSBPL7 | 0.340934 | 0.987665 | 0.962756 | 1.013219 |  |
| AGK | 0.795564 | 0.996572 | 0.971016 | 1.0228 |  |
| ALDH3B1 | 0.578548 | 1.000614 | 0.998448 | 1.002785 |  |
| ARSD | 0.89763 | 1.000212 | 0.996982 | 1.003453 |  |
| PNPLA4 | 0.775796 | 1.002237 | 0.986945 | 1.017766 |  |
| STARD3NL | 0.170607 | 1.005748 | 0.997536 | 1.014027 |  |
| ABHD5 | 0.233357 | 1.011169 | 0.992866 | 1.029809 |  |
| PIK3C2A | 0.553345 | 0.996326 | 0.984274 | 1.008526 |  |
| MBTPS2 | 0.00154 | 1.045728 | 1.017189 | 1.075067 |  |
| ELOVL5 | 0.327822 | 1.001116 | 0.998882 | 1.003354 |  |
| ALOX5 | 0.184902 | 0.996694 | 0.991826 | 1.001585 |  |
| DPEP1 | 0.45122 | 0.995699 | 0.984598 | 1.006926 |  |
| AGPS | 0.01212 | 1.014692 | 1.003195 | 1.026321 |  |
| CYP24A1 | 0.00928 | 1.000689 | 1.00017 | 1.001208 |  |
| OSBPL5 | 0.117605 | 1.009889 | 0.997519 | 1.022413 |  |
| AGPAT4 | 0.780322 | 1.008436 | 0.950626 | 1.069762 |  |
| PNPLA6 | 0.114402 | 0.990751 | 0.979386 | 1.002247 |  |
| CYP46A1 | 0.150064 | 0.732129 | 0.47884 | 1.119398 |  |
| PI4K2B | 0.297776 | 0.995398 | 0.986792 | 1.004078 |  |
| INPP4A | 0.218862 | 0.978125 | 0.944241 | 1.013225 |  |
| HEXB | 0.60085 | 1.000988 | 0.997292 | 1.004697 |  |
| PIK3CB | 0.221849 | 1.007585 | 0.995443 | 1.019876 |  |
| PLEKHA5 | 0.646049 | 1.004041 | 0.986908 | 1.021471 |  |
| MSMO1 | 0.00366 | 1.003683 | 1.001197 | 1.006174 |  |
| GALC | 0.641205 | 1.002692 | 0.99142 | 1.014092 |  |
| TBXAS1 | 0.559399 | 0.99552 | 0.980626 | 1.010641 |  |
| ACAA1 | 0.184861 | 0.987765 | 0.969953 | 1.005904 |  |
| ARSF | 0.017369 | 2.812278 | 1.199681 | 6.592513 |  |
| DGAT2 | 0.551634 | 0.99086 | 0.961356 | 1.02127 |  |
| SPHK2 | 0.503843 | 0.987356 | 0.951198 | 1.024889 |  |
| MTMR1 | 0.61336 | 0.995691 | 0.979151 | 1.01251 |  |
| CTSA | 0.672328 | 0.999601 | 0.997753 | 1.001452 |  |
| FAR2 | 0.099667 | 1.018797 | 0.99646 | 1.041635 |  |
| ELOVL1 | 0.199007 | 1.001569 | 0.999176 | 1.003968 |  |
| IDI1 | 0.138388 | 1.003263 | 0.99895 | 1.007594 |  |
| PPAP2A | 0.163834 | 0.996971 | 0.992723 | 1.001238 |  |
| ACSL4 | 0.061108 | 1.003412 | 0.999841 | 1.006996 |  |
| PLA2G10 | 0.656394 | 0.99899 | 0.994552 | 1.003447 |  |
| SLC44A1 | 0.014078 | 1.007213 | 1.001451 | 1.013009 |  |
| GBA2 | 0.653074 | 0.998021 | 0.989437 | 1.00668 |  |
| CSNK2A2 | 0.222729 | 1.007854 | 0.995259 | 1.020608 |  |
| OSBPL3 | 0.248772 | 1.006904 | 0.995199 | 1.018747 |  |
| PRKACA | 0.347094 | 0.99326 | 0.979355 | 1.007362 |  |
| ALDH3A2 | 0.188702 | 0.9988 | 0.997013 | 1.00059 |  |
| ACADVL | 0.526882 | 0.99932 | 0.997218 | 1.001427 |  |
| ABCB11 | 0.300908 | 1.250908 | 0.818503 | 1.911748 |  |
| PTGS2 | 0.921035 | 0.999936 | 0.998665 | 1.001208 |  |
| MGLL | 0.453304 | 0.998638 | 0.995087 | 1.002202 |  |
| PTPLAD1 | 0.62117 | 1.001107 | 0.996725 | 1.005507 |  |
| ACAT1 | 0.128544 | 0.993856 | 0.985989 | 1.001786 |  |
| PLD1 | 0.332227 | 1.017636 | 0.982303 | 1.054241 |  |
| ACACB | 0.269154 | 0.958447 | 0.888945 | 1.033382 |  |
| ACER3 | 0.775334 | 0.996637 | 0.973849 | 1.019959 |  |
| PIK3C3 | 0.309204 | 0.977335 | 0.93509 | 1.021489 |  |
| SYNJ2 | 0.175928 | 1.016207 | 0.99282 | 1.040145 |  |
| OSBPL6 | 0.050752 | 0.928286 | 0.861506 | 1.000243 |  |
| LIPE | 0.904257 | 0.996572 | 0.942346 | 1.053918 |  |
| LRP2 | 0.286219 | 0.989014 | 0.969138 | 1.009298 |  |
| AACS | 0.369369 | 1.011612 | 0.98643 | 1.037436 |  |
| OXCT1 | 0.43086 | 1.002764 | 0.9959 | 1.009676 |  |
| SLC27A5 | 0.319345 | 1.060041 | 0.945109 | 1.18895 |  |
| STARD7 | 0.674065 | 1.000747 | 0.997272 | 1.004234 |  |
| SLCO1A2 | 0.542852 | 1.027647 | 0.941229 | 1.121998 |  |
| HADHA | 0.03124 | 1.002789 | 1.000251 | 1.005334 |  |
| AKR1B1 | 0.60391 | 1.000351 | 0.999026 | 1.001678 |  |
| CPNE3 | 0.252976 | 1.00246 | 0.998246 | 1.006692 |  |
| DDHD2 | 0.167904 | 1.005712 | 0.997603 | 1.013886 |  |
| HSD17B2 | 0.004505 | 1.020855 | 1.006419 | 1.035498 |  |
| ACOX3 | 0.66194 | 0.995934 | 0.977909 | 1.014291 |  |
| MTMR2 | 0.00084 | 1.019971 | 1.008201 | 1.031877 |  |
| HSD17B14 | 0.325513 | 0.997694 | 0.993113 | 1.002296 |  |
| ACHE | 0.710948 | 0.998987 | 0.993648 | 1.004355 |  |
| PGS1 | 0.022523 | 0.981947 | 0.966699 | 0.997436 |  |
| LPCAT2 | 0.122807 | 0.984661 | 0.965517 | 1.004185 |  |
| CRLS1 | 0.289022 | 0.996784 | 0.990868 | 1.002737 |  |
| SPTLC1 | 0.020448 | 1.0089 | 1.00137 | 1.016487 |  |
| CERS4 | 0.003002 | 0.990888 | 0.984916 | 0.996897 |  |
| PITPNM2 | 0.351927 | 1.012919 | 0.985909 | 1.040669 |  |
| OSBPL8 | 0.942048 | 0.999633 | 0.989791 | 1.009573 |  |
| PITPNM3 | 0.281865 | 0.979379 | 0.942909 | 1.01726 |  |
| PTGS1 | 0.247621 | 1.006144 | 0.995751 | 1.016645 |  |
| CRAT | 0.913821 | 0.999514 | 0.990745 | 1.00836 |  |
| ACOT7 | 0.000219 | 1.009631 | 1.004512 | 1.014775 |  |
| SCD | 0.178319 | 1.000397 | 0.999819 | 1.000975 |  |
| HSD3B7 | 0.210088 | 1.004763 | 0.997324 | 1.012257 |  |
| TECR | 0.975674 | 0.999879 | 0.992159 | 1.007659 |  |
| GGT5 | 0.274699 | 0.995109 | 0.986394 | 1.003902 |  |
| GGT1 | 0.837035 | 0.999084 | 0.990394 | 1.007849 |  |
| SLC25A1 | 0.030315 | 1.002514 | 1.000239 | 1.004794 |  |
| PLA2G3 | 0.114314 | 0.971414 | 0.937067 | 1.00702 |  |
| CYP2D6 | 0.125488 | 0.967479 | 0.927414 | 1.009275 |  |
| SBF1 | 0.689649 | 0.998214 | 0.989494 | 1.007011 |  |
| CHKB | 0.050105 | 0.956822 | 0.91549 | 1.00002 |  |
| MCAT | 0.057293 | 1.018944 | 0.999419 | 1.038851 |  |
| ARSA | 0.369777 | 0.997942 | 0.993459 | 1.002445 |  |
| TSPO | 0.166647 | 1.000869 | 0.999638 | 1.002102 |  |
| MTMR3 | 0.208268 | 0.953472 | 0.885276 | 1.026922 |  |
| PNPLA5 | 0.902505 | 1.004366 | 0.936748 | 1.076864 |  |
| PNPLA3 | 0.008325 | 1.108404 | 1.026824 | 1.196465 |  |
| SLC25A17 | 0.007243 | 1.020335 | 1.005453 | 1.035437 |  |
| CERK | 0.460202 | 0.996759 | 0.988209 | 1.005383 |  |
| ABHD4 | 0.013652 | 1.006481 | 1.001327 | 1.011662 |  |
| DDHD1 | 0.132163 | 0.952434 | 0.893887 | 1.014815 |  |
| SPTLC2 | 0.180827 | 0.993777 | 0.984732 | 1.002905 |  |
| LGMN | 0.495966 | 0.999511 | 0.998105 | 1.000919 |  |
| SLC10A1 | 0.302428 | 0.878934 | 0.687766 | 1.123239 |  |
| CPNE6 | 0.283319 | 0.476631 | 0.123108 | 1.845352 |  |
| SEC23A | 0.045377 | 1.005405 | 1.000111 | 1.010727 |  |
| CSNK2A1 | 0.136502 | 1.004407 | 0.99861 | 1.010238 |  |
| CDS2 | 0.901598 | 0.998929 | 0.982104 | 1.016043 |  |
| ACOT8 | 0.266264 | 0.988218 | 0.967783 | 1.009084 |  |
| VAPA | 0.771435 | 0.999246 | 0.994172 | 1.004346 |  |
| LPIN2 | 0.306092 | 0.998516 | 0.995679 | 1.00136 |  |
| STS | 0.365441 | 0.996544 | 0.9891 | 1.004044 |  |
| ABCD1 | 0.000882 | 1.014638 | 1.005985 | 1.023365 |  |
| BMX | 0.710411 | 0.997707 | 0.985688 | 1.009872 |  |
| MTMR8 | 0.058717 | 0.803894 | 0.641069 | 1.008076 |  |
| TAZ | 0.534631 | 0.99471 | 0.978196 | 1.011503 |  |
| PCYT1B | 0.805687 | 0.985667 | 0.878565 | 1.105824 |  |
| GLA | 0.030909 | 1.004459 | 1.000409 | 1.008526 |  |
| GDPD3 | 0.657473 | 1.001356 | 0.995376 | 1.007372 |  |
| VAC14 | 0.490316 | 0.994651 | 0.979608 | 1.009925 |  |
| SMPD3 | 0.805165 | 1.001831 | 0.987377 | 1.016495 |  |
| PLA2G15 | 0.287189 | 0.991814 | 0.976914 | 1.006941 |  |
| FA2H | 0.070115 | 1.002743 | 0.999775 | 1.005719 |  |
| MLYCD | 0.027507 | 0.871841 | 0.771747 | 0.984915 |  |
| ABCC1 | 0.003008 | 1.005405 | 1.001831 | 1.008991 |  |
| UBE2I | 0.104755 | 1.010078 | 0.997915 | 1.022389 |  |
| CDIPT | 0.469175 | 0.998226 | 0.993438 | 1.003037 |  |
| ACSBG1 | 0.02611 | 0.5383 | 0.31192 | 0.928981 |  |
| DECR1 | 0.009564 | 1.009034 | 1.002193 | 1.015921 |  |
| SQLE | 0.847211 | 1.000171 | 0.998436 | 1.001908 |  |
| MTMR9 | 0.948065 | 0.998187 | 0.945144 | 1.054207 |  |
| ASAH1 | 0.252031 | 0.999363 | 0.998273 | 1.000453 |  |
| LHB | 0.282658 | 1.032861 | 0.9737 | 1.095616 |  |
| PLD3 | 0.310551 | 0.999668 | 0.999026 | 1.00031 |  |
| PIAS4 | 0.803743 | 1.002944 | 0.97996 | 1.026466 |  |
| PRKD2 | 0.079854 | 1.010053 | 0.99881 | 1.021423 |  |
| PLIN3 | 3.93E-05 | 1.004863 | 1.002542 | 1.007189 |  |
| PLA2G4C | 0.930745 | 1.00239 | 0.949848 | 1.057839 |  |
| PLEKHA4 | 0.69159 | 0.997612 | 0.985896 | 1.009467 |  |
| PIK3R2 | 0.182181 | 0.615307 | 0.301462 | 1.255886 |  |
| PIK3CG | 0.003074 | 0.934743 | 0.893896 | 0.977455 |  |
| PON3 | 0.430575 | 0.998734 | 0.995592 | 1.001885 |  |
| PON2 | 0.336199 | 1.000563 | 0.999416 | 1.001712 |  |
| CAV1 | 0.007335 | 1.000805 | 1.000216 | 1.001393 |  |
| PLEKHA8 | 0.96423 | 1.000674 | 0.971648 | 1.030566 |  |
| PRKAG2 | 0.14255 | 0.977844 | 0.948994 | 1.007571 |  |
| PTGR1 | 0.007386 | 1.001376 | 1.000369 | 1.002385 |  |
| PIP5K1B | 0.3647 | 0.989376 | 0.966788 | 1.012492 |  |
| PTGDS | 0.010036 | 0.997271 | 0.995199 | 0.999348 |  |
| PHYH | 0.559719 | 0.997703 | 0.990021 | 1.005444 |  |
| CUBN | 0.137206 | 0.795406 | 0.588159 | 1.07568 |  |
| PLEKHA1 | 0.473638 | 0.988176 | 0.956543 | 1.020854 |  |
| ACBD5 | 0.005154 | 1.012006 | 1.003579 | 1.020503 |  |
| MTMR4 | 0.263538 | 0.99178 | 0.977532 | 1.006235 |  |
| KPNB1 | 0.014642 | 1.00342 | 1.000673 | 1.006174 |  |
| HSD17B1 | 0.734718 | 0.979297 | 0.867686 | 1.105265 |  |
| ALOX12 | 0.321987 | 0.962086 | 0.89124 | 1.038564 |  |
| ABCC3 | 0.459195 | 0.999171 | 0.996979 | 1.001368 |  |
| DHRS7B | 0.642903 | 0.995108 | 0.974689 | 1.015954 |  |
| INPP4B | 0.211132 | 1.011303 | 0.993643 | 1.029277 |  |
| SC5D | 0.180174 | 0.997607 | 0.994118 | 1.001108 |  |
| OSBP | 0.422291 | 1.003207 | 0.995391 | 1.011085 |  |
| PTPMT1 | 0.775274 | 0.997868 | 0.983353 | 1.012598 |  |
| PITPNM1 | 0.259228 | 1.004131 | 0.996965 | 1.011349 |  |
| CHKA | 0.004175 | 0.987347 | 0.978781 | 0.995987 |  |
| MVK | 0.544819 | 0.990851 | 0.961809 | 1.02077 |  |
| PTGES3 | 0.001207 | 1.001081 | 1.000426 | 1.001736 |  |
| CYP27B1 | 0.858984 | 1.001565 | 0.984437 | 1.018991 |  |
| ACSS3 | 0.066038 | 0.943397 | 0.886568 | 1.003868 |  |
| LTA4H | 0.139326 | 0.997775 | 0.994832 | 1.000726 |  |
| ACAD10 | 0.570665 | 0.991519 | 0.962756 | 1.021141 |  |
| VDR | 0.639271 | 1.001657 | 0.994748 | 1.008613 |  |
| CHPT1 | 0.049811 | 1.007919 | 1.000007 | 1.015893 |  |
| LPCAT3 | 0.328597 | 0.990072 | 0.97045 | 1.01009 |  |
| SLCO1B3 | 0.046743 | 1.016146 | 1.000231 | 1.032314 |  |
| PPARD | 0.020879 | 1.006301 | 1.000953 | 1.011678 |  |
| ACOT13 | 0.503899 | 1.0038 | 0.992698 | 1.015026 |  |
| FIG4 | 0.404161 | 0.990453 | 0.96838 | 1.013029 |  |
| COL4A3BP | 0.100153 | 0.985358 | 0.968184 | 1.002838 |  |
| ARSB | 0.106848 | 1.026059 | 0.994473 | 1.058648 |  |
| SEC24A | 0.319539 | 1.005922 | 0.994295 | 1.017686 |  |
| EHHADH | 0.006433 | 1.025358 | 1.007054 | 1.043994 |  |
| PCCB | 0.370222 | 1.006229 | 0.992654 | 1.019989 |  |
| BCHE | 0.396464 | 1.004212 | 0.994503 | 1.014017 |  |
| SCAP | 0.976665 | 1.000157 | 0.989688 | 1.010736 |  |
| PIKFYVE | 0.338862 | 0.98764 | 0.962786 | 1.013135 |  |
| POMC | 0.464498 | 0.988692 | 0.959018 | 1.019284 |  |
| GPD2 | 0.062224 | 1.008001 | 0.999593 | 1.016481 |  |
| ACADL | 0.098202 | 0.957629 | 0.909728 | 1.008052 |  |
| PECR | 0.411167 | 1.005829 | 0.991984 | 1.019867 |  |
| PRKD3 | 0.342483 | 0.991803 | 0.975091 | 1.008802 |  |
| PLEKHA3 | 0.249487 | 0.963586 | 0.904634 | 1.02638 |  |
| DHCR24 | 0.229773 | 0.999687 | 0.999175 | 1.000198 |  |
| SCP2 | 0.551813 | 1.002156 | 0.995072 | 1.00929 |  |
| MECR | 0.793036 | 1.003407 | 0.978233 | 1.02923 |  |
| PLA2G4A | 0.950585 | 0.999949 | 0.99835 | 1.001551 |  |
| HAO2 | 0.554711 | 0.660731 | 0.167061 | 2.613207 |  |
| GNPAT | 0.021774 | 1.006919 | 1.001005 | 1.012867 |  |
| PLA2G2D | 0.896072 | 0.999449 | 0.991219 | 1.007747 |  |
| HMGCL | 0.84875 | 1.001228 | 0.988675 | 1.013941 |  |
| PIK3R3 | 0.30332 | 0.993611 | 0.981558 | 1.005812 |  |
| FAAH | 0.014748 | 0.987082 | 0.97682 | 0.997452 |  |
| HSD11B1 | 0.771374 | 1.001455 | 0.991679 | 1.011328 |  |
| OSBPL9 | 0.988417 | 0.99993 | 0.990482 | 1.009468 |  |
| ESYT2 | 0.829684 | 1.000243 | 0.998034 | 1.002457 |  |
| ELOVL4 | 0.859426 | 1.002683 | 0.973391 | 1.032856 |  |
| RAB14 | 0.880393 | 1.000335 | 0.995985 | 1.004703 |  |
| KDSR | 0.273859 | 1.011078 | 0.991321 | 1.031228 |  |
| ACOT2 | 0.823769 | 1.001737 | 0.986556 | 1.017151 |  |
| ELOVL3 | 0.026801 | 1.013204 | 1.001508 | 1.025036 |  |
| GPAM | 0.038616 | 0.970005 | 0.942411 | 0.998407 |  |
| ACAT2 | 0.00086 | 1.017185 | 1.007043 | 1.027428 |  |
| EPHX2 | 0.058376 | 0.978319 | 0.956364 | 1.000777 |  |
| PLBD1 | 7.98E-05 | 1.004253 | 1.002138 | 1.006373 |  |
| FABP3 | 0.368969 | 0.999392 | 0.998067 | 1.000719 |  |
| PIK3CA | 0.24662 | 1.013873 | 0.990516 | 1.03778 |  |
| OCRL | 0.38176 | 1.00479 | 0.994088 | 1.015608 |  |
| AKR1D1 | 0.528169 | 0.701327 | 0.232906 | 2.111835 |  |
| ACADS | 0.839412 | 0.999136 | 0.990822 | 1.007521 |  |
| ACOT9 | 0.070695 | 1.014212 | 0.99881 | 1.029852 |  |
| ORMDL2 | 0.014791 | 1.009408 | 1.001836 | 1.017037 |  |
| LPGAT1 | 0.000134 | 1.010722 | 1.005206 | 1.016269 |  |
| PLA2G12A | 0.777869 | 1.002107 | 0.98756 | 1.016868 |  |
| ACSL3 | 0.002535 | 1.005973 | 1.002091 | 1.00987 |  |
| MOGAT1 | 0.747859 | 1.0793 | 0.677757 | 1.718742 |  |
| VAPB | 0.62786 | 0.996594 | 0.982941 | 1.010437 |  |
| PTGIS | 0.027595 | 1.002086 | 1.00023 | 1.003946 |  |
| MCEE | 0.546671 | 0.994898 | 0.978488 | 1.011584 |  |
| SLC10A2 | 0.002822 | 1.011115 | 1.003806 | 1.018476 |  |
| MBOAT7 | 0.062932 | 1.00475 | 0.999744 | 1.00978 |  |
| INSIG2 | 0.069823 | 0.990493 | 0.980317 | 1.000774 |  |
| GPCPD1 | 0.413641 | 0.998004 | 0.993233 | 1.002797 |  |
| SGPP1 | 0.170713 | 1.003245 | 0.998606 | 1.007906 |  |
| HRASLS | 0.24075 | 1.012624 | 0.991621 | 1.034072 |  |
| PLA2G5 | 0.339023 | 0.960039 | 0.883044 | 1.043749 |  |
| ECHS1 | 0.559682 | 1.00049 | 0.998845 | 1.002137 |  |
| SRD5A3 | 0.870279 | 1.000222 | 0.997565 | 1.002885 |  |
| ORMDL1 | 0.186177 | 0.990684 | 0.977032 | 1.004527 |  |
| SUMF2 | 0.389032 | 1.00041 | 0.999478 | 1.001343 |  |
| PLD2 | 0.805063 | 1.002199 | 0.984867 | 1.019837 |  |
| SLC44A2 | 0.695788 | 1.000449 | 0.998199 | 1.002704 |  |
| ACSBG2 | 0.396405 | 0.854831 | 0.594904 | 1.228325 |  |
| CYP2E1 | 0.551349 | 0.960309 | 0.840511 | 1.097181 |  |
| PNPLA7 | 0.495206 | 0.990226 | 0.962668 | 1.018573 |  |
| OSBPL2 | 0.209314 | 0.989528 | 0.973399 | 1.005925 |  |
| HSD17B3 | 0.231617 | 0.89981 | 0.756881 | 1.06973 |  |
| PPT1 | 0.420401 | 0.999397 | 0.997933 | 1.000864 |  |
| HACL1 | 0.949025 | 1.000886 | 0.974082 | 1.028427 |  |
| ACLY | 0.716991 | 1.000377 | 0.998341 | 1.002417 |  |
| STARD3 | 0.673199 | 0.998798 | 0.993229 | 1.004397 |  |
| PRKAB2 | 0.217923 | 1.009478 | 0.994443 | 1.02474 |  |
| HSD17B7 | 0.283728 | 0.984192 | 0.955933 | 1.013287 |  |
| RAN | 0.003686 | 1.00231 | 1.00075 | 1.003872 |  |
| INPP5K | 0.351144 | 0.991794 | 0.974764 | 1.009122 |  |
| ALDH3B2 | 0.924705 | 0.999722 | 0.993972 | 1.005505 |  |
| LPIN3 | 0.783883 | 0.997923 | 0.983207 | 1.01286 |  |
| TPTE2 | 0.311141 | 0.80309 | 0.525377 | 1.227603 |  |
| ALOX5AP | 0.142201 | 0.998192 | 0.995782 | 1.000607 |  |
| PEMT | 0.837988 | 1.000621 | 0.994683 | 1.006595 |  |
| PIK3C2B | 0.065972 | 1.013488 | 0.999116 | 1.028066 |  |
| CSNK1G2 | 0.702954 | 1.001604 | 0.993388 | 1.009888 |  |
| RARRES3 | 0.558809 | 1.00012 | 0.999717 | 1.000523 |  |
| HRASLS2 | 0.413046 | 1.001625 | 0.997739 | 1.005526 |  |
| MORC2 | 0.122054 | 1.009286 | 0.99753 | 1.02118 |  |
| SBF2 | 0.308586 | 1.017697 | 0.983905 | 1.05265 |  |
| HSD17B4 | 0.064217 | 0.993212 | 0.986072 | 1.000402 |  |
| CEPT1 | 0.457403 | 0.987722 | 0.956057 | 1.020435 |  |
| ARF3 | 0.039651 | 1.001729 | 1.000082 | 1.003378 |  |
| LPIN1 | 0.265874 | 0.989282 | 0.970681 | 1.00824 |  |
| CYP2J2 | 0.64673 | 1.005343 | 0.98269 | 1.028519 |  |
| FADS2 | 0.132434 | 1.003389 | 0.998976 | 1.007821 |  |
| PNPLA8 | 0.443209 | 1.005477 | 0.991537 | 1.019613 |  |
| HILPDA | 0.802377 | 0.999775 | 0.998015 | 1.001538 |  |
| CGA | 0.638998 | 1.000121 | 0.999616 | 1.000626 |  |
| B4GALNT1 | 0.03105 | 1.027029 | 1.002433 | 1.052228 |  |
| SMPD2 | 0.721528 | 0.996685 | 0.978654 | 1.01505 |  |
| CYP27A1 | 0.004738 | 0.995235 | 0.991942 | 0.998539 |  |
| SMPD4 | 0.013492 | 1.011981 | 1.002464 | 1.021589 |  |
| BAAT | 0.667171 | 0.996673 | 0.981651 | 1.011925 |  |
| FDX1 | 0.155886 | 1.002829 | 0.998924 | 1.006749 |  |
| CYP19A1 | 0.693622 | 0.951402 | 0.74254 | 1.219012 |  |
| SLC44A5 | 0.636559 | 0.996734 | 0.9833 | 1.010352 |  |
| EPT1 | 0.018626 | 1.008019 | 1.001335 | 1.014747 |  |
| HADHB | 0.48415 | 1.001982 | 0.996439 | 1.007557 |  |
| CYP1B1 | 0.987103 | 1.000015 | 0.998182 | 1.001852 |  |
| CYP2C9 | 0.924175 | 0.997824 | 0.954048 | 1.043608 |  |
| CYP2C8 | 0.221628 | 0.903822 | 0.768523 | 1.062941 |  |
| CH25H | 0.073374 | 0.995388 | 0.990365 | 1.000438 |  |
| AGPAT9 | 1.65E-05 | 1.014124 | 1.007674 | 1.020615 |  |
| HADH | 0.344662 | 0.997253 | 0.991578 | 1.002959 |  |
| SEC24B | 0.578662 | 1.003771 | 0.990524 | 1.017195 |  |
| PIK3C2G | 0.428961 | 0.989159 | 0.962799 | 1.01624 |  |
| ETNK1 | 0.159067 | 1.00425 | 0.99834 | 1.010195 |  |
| GLTP | 0.490191 | 0.998492 | 0.99422 | 1.002782 |  |
| MTMR6 | 0.337341 | 0.992037 | 0.975966 | 1.008372 |  |
| CERS5 | 0.813666 | 1.002777 | 0.979917 | 1.026171 |  |
| ESYT1 | 0.424903 | 1.001904 | 0.997234 | 1.006596 |  |
| FITM1 | 0.041953 | 0.723455 | 0.52959 | 0.988289 |  |
| PTGR2 | 0.366741 | 1.027015 | 0.969255 | 1.088216 |  |
| SLC27A2 | 0.722079 | 1.00131 | 0.994112 | 1.00856 |  |
| CYP11A1 | 0.512 | 1.002708 | 0.994635 | 1.010846 |  |
| CYP1A2 | 0.834042 | 1.004499 | 0.963194 | 1.047574 |  |
| MBTPS1 | 0.490252 | 0.997242 | 0.989448 | 1.005097 |  |
| DPEP3 | 0.254034 | 1.00951 | 0.993224 | 1.026064 |  |
| PCTP | 0.946106 | 0.999424 | 0.982861 | 1.016266 |  |
| ARSG | 0.070737 | 0.911012 | 0.823428 | 1.007912 |  |
| OSBPL1A | 0.434026 | 0.994648 | 0.981364 | 1.008111 |  |
| PIK3R5 | 0.09523 | 0.969299 | 0.934436 | 1.005463 |  |
| PPAP2C | 0.510463 | 0.997964 | 0.991926 | 1.004039 |  |
| PRKACB | 0.343087 | 1.002333 | 0.997516 | 1.007174 |  |
| CYP4B1 | 0.003677 | 0.99823 | 0.997037 | 0.999424 |  |
| SLC44A3 | 0.761032 | 0.998726 | 0.990555 | 1.006964 |  |
| PI4KB | 0.456675 | 0.997261 | 0.990083 | 1.00449 |  |
| PIP5K1A | 0.942123 | 0.999774 | 0.993681 | 1.005903 |  |
| CERS2 | 0.600823 | 0.999513 | 0.997692 | 1.001338 |  |
| SLC27A3 | 0.216137 | 0.993183 | 0.982476 | 1.004007 |  |
| DEGS1 | 0.084842 | 1.002702 | 0.999629 | 1.005784 |  |
| ARF1 | 0.179034 | 1.000476 | 0.999782 | 1.001171 |  |
| MBOAT2 | 0.030909 | 1.006877 | 1.00063 | 1.013163 |  |
| LBR | 0.003384 | 1.007818 | 1.002582 | 1.013081 |  |
| ETNK2 | 0.520669 | 1.006104 | 0.987593 | 1.024963 |  |
| PLEKHA6 | 0.006862 | 1.009794 | 1.002684 | 1.016955 |  |
| SUMF1 | 0.354527 | 1.006023 | 0.993316 | 1.018892 |  |
| RAB5A | 0.339894 | 1.003728 | 0.996087 | 1.011427 |  |
| OSBPL10 | 0.243315 | 1.018754 | 0.987447 | 1.051053 |  |
| PLA1A | 0.182146 | 0.994864 | 0.987367 | 1.002418 |  |
| SCD5 | 0.933859 | 0.999541 | 0.988767 | 1.010433 |  |
| CBR4 | 0.4785 | 0.990708 | 0.965459 | 1.016618 |  |
| SRD5A1 | 0.355685 | 1.006869 | 0.992348 | 1.021602 |  |
| PIK3R1 | 0.139025 | 0.985544 | 0.966714 | 1.004741 |  |
| TNFAIP8 | 0.857499 | 0.997902 | 0.975282 | 1.021046 |  |
| MUT | 0.62374 | 0.997719 | 0.988659 | 1.006862 |  |
| HMGCLL1 | 0.00999 | 0.752752 | 0.606469 | 0.934319 |  |
| CYP39A1 | 0.3826 | 0.986199 | 0.955909 | 1.017449 |  |
| EBP | 0.12792 | 1.002789 | 0.9992 | 1.006392 |  |
| AWAT2 | 0.907446 | 0.982746 | 0.732844 | 1.317865 |  |
| NSDHL | 0.007575 | 1.008415 | 1.002232 | 1.014636 |  |
| STAR | 0.431499 | 0.816021 | 0.491695 | 1.354273 |  |
| UGCG | 0.247102 | 1.002467 | 0.998293 | 1.006659 |  |
| PTGES2 | 0.353189 | 1.003452 | 0.996179 | 1.010778 |  |
| FAM73B | 0.1224 | 0.975987 | 0.946347 | 1.006554 |  |
| PTGES | 5.17E-05 | 1.001965 | 1.001013 | 1.002918 |  |
| INPP5E | 0.417176 | 0.990889 | 0.969221 | 1.013042 |  |
| CYP17A1 | 0.011264 | 0.627912 | 0.438132 | 0.899895 |  |
| HSD17B12 | 0.808575 | 1.001242 | 0.991239 | 1.011346 |  |
| TM7SF2 | 0.705053 | 1.001221 | 0.994915 | 1.007567 |  |
| MTMR12 | 0.093013 | 0.99179 | 0.982295 | 1.001376 |  |
| PIP4K2A | 0.469467 | 1.0028 | 0.995231 | 1.010427 |  |
| SEC24D | 0.744519 | 0.9983 | 0.988137 | 1.008568 |  |
| THRSP | 0.173966 | 1.195109 | 0.924307 | 1.545249 |  |
| MMAA | 0.698672 | 0.983343 | 0.903169 | 1.070632 |  |
| AKR1C2 | 0.391446 | 1.000155 | 0.9998 | 1.00051 |  |
| OLAH | 0.026071 | 1.048717 | 1.005685 | 1.09359 |  |
| GPD1L | 0.000605 | 0.99038 | 0.984924 | 0.995866 |  |
| SAR1B | 0.320026 | 0.99061 | 0.972361 | 1.009202 |  |
| GGPS1 | 0.205208 | 1.007425 | 0.995961 | 1.019021 |  |
| ACOXL | 0.002948 | 0.916208 | 0.864846 | 0.97062 |  |
| PLA2R1 | 0.621464 | 0.984774 | 0.926587 | 1.046614 |  |
| LPCAT1 | 0.322734 | 0.999787 | 0.999365 | 1.000209 |  |
| GDPD1 | 0.071169 | 0.974045 | 0.946615 | 1.00227 |  |
| CERS3 | 0.654363 | 0.980599 | 0.9 | 1.068415 |  |
| CYP2U1 | 0.020857 | 0.940366 | 0.892577 | 0.990714 |  |
| AGPAT5 | 0.00168 | 1.015864 | 1.005937 | 1.025888 |  |
| PI4K2A | 0.695945 | 1.002743 | 0.989061 | 1.016615 |  |
| DBI | 0.495005 | 0.999207 | 0.996933 | 1.001486 |  |
| PTDSS1 | 0.000299 | 1.013458 | 1.006141 | 1.020829 |  |
| SAMD8 | 0.82436 | 1.002252 | 0.982541 | 1.022358 |  |
| ARSE | 0.613868 | 1.000813 | 0.997657 | 1.00398 |  |
| FAM213B | 0.84813 | 1.000691 | 0.993644 | 1.007787 |  |
| ABHD3 | 0.151108 | 1.005938 | 0.99784 | 1.014102 |  |
| ESYT3 | 0.001281 | 0.947494 | 0.916895 | 0.979114 |  |
| GDPD5 | 0.803339 | 0.995825 | 0.963565 | 1.029164 |  |
| AGPAT6 | 0.838403 | 0.999324 | 0.992848 | 1.005842 |  |
| PLA2G2F | 0.653443 | 1.041285 | 0.872716 | 1.242412 |  |
| SYNJ1 | 0.137416 | 0.968362 | 0.928146 | 1.010322 |  |
| CBR1 | 0.285934 | 0.99984 | 0.999545 | 1.000134 |  |
| PLA2G4D | 0.996711 | 1.000242 | 0.891374 | 1.122407 |  |
| THEM4 | 0.909905 | 1.000923 | 0.98506 | 1.017041 |  |
| AGPAT3 | 0.610977 | 1.003097 | 0.991217 | 1.015118 |  |
| LSS | 0.619225 | 0.997409 | 0.987256 | 1.007667 |  |
| FDPS | 0.004294 | 1.00425 | 1.001331 | 1.007177 |  |
| CYP3A4 | 0.679426 | 0.878759 | 0.47605 | 1.622136 |  |
| PCYT1A | 0.02712 | 1.015187 | 1.001706 | 1.02885 |  |
| BDH1 | 0.683881 | 1.005075 | 0.980879 | 1.029868 |  |
| FDXR | 0.674884 | 0.99629 | 0.979137 | 1.013744 |  |
| ALOX15 | 0.518754 | 1.001487 | 0.996978 | 1.006016 |  |
| NEU3 | 0.423552 | 0.978791 | 0.928727 | 1.031553 |  |
| CYP4A22 | 0.196806 | 0.558053 | 0.230142 | 1.353178 |  |
| ACOT11 | 0.154692 | 1.057741 | 0.979048 | 1.142758 |  |
| PPAP2B | 0.995147 | 1.000023 | 0.992707 | 1.007393 |  |
| PRKAA2 | 0.47576 | 1.00978 | 0.983129 | 1.037153 |  |
| ACP6 | 0.182749 | 1.021862 | 0.989864 | 1.054895 |  |
| MAPKAPK2 | 0.733418 | 1.000591 | 0.997195 | 1.003998 |  |
| SGPP2 | 0.918187 | 1.000115 | 0.997926 | 1.002309 |  |
| HPGDS | 0.018142 | 0.970248 | 0.94624 | 0.994865 |  |
| TNFAIP8L2 | 0.05062 | 0.987503 | 0.97513 | 1.000034 |  |
| PMVK | 0.611003 | 0.999345 | 0.996825 | 1.001871 |  |
| GLB1L | 0.353906 | 0.989452 | 0.967517 | 1.011885 |  |
| PPM1L | 0.129093 | 1.030075 | 0.991402 | 1.070257 |  |
| CDS1 | 0.654105 | 0.998 | 0.989301 | 1.006777 |  |
| PTPN13 | 0.558419 | 0.998733 | 0.994502 | 1.002982 |  |
| ALB | 0.378084 | 0.998184 | 0.994159 | 1.002226 |  |
| MTMR14 | 0.941043 | 0.999244 | 0.97941 | 1.019479 |  |
| PLB1 | 0.109609 | 1.056224 | 0.987767 | 1.129426 |  |
| LIPH | 0.005195 | 1.002814 | 1.00084 | 1.004792 |  |
| SGMS2 | 0.665728 | 0.998566 | 0.992089 | 1.005087 |  |
| BDH2 | 0.012893 | 0.982367 | 0.968688 | 0.996239 |  |
| ETNPPL | 0.445541 | 0.992725 | 0.974276 | 1.011524 |  |
| HPGD | 0.363828 | 0.999599 | 0.998733 | 1.000465 |  |
| ELOVL7 | 0.099709 | 1.013899 | 0.997373 | 1.030699 |  |
| STARD4 | 0.00341 | 1.023048 | 1.007563 | 1.038772 |  |
| ARSK | 0.403945 | 1.022652 | 0.970249 | 1.077885 |  |
| ENPP6 | 0.109013 | 0.608464 | 0.331408 | 1.117136 |  |
| ACSL6 | 0.455608 | 0.890506 | 0.656652 | 1.207642 |  |
| FABP7 | 0.06761 | 1.000705 | 0.999949 | 1.001461 |  |
| FABP5 | 0.733285 | 1.000848 | 0.995978 | 1.005743 |  |
| MID1IP1 | 0.370775 | 0.998193 | 0.994248 | 1.002154 |  |
| SPTSSA | 0.938453 | 1.000036 | 0.999128 | 1.000944 |  |
| INPPL1 | 0.300957 | 0.998045 | 0.994352 | 1.001752 |  |
| FAAH2 | 0.354955 | 1.004758 | 0.994703 | 1.014915 |  |
| TMEM55B | 0.188127 | 0.990365 | 0.97619 | 1.004747 |  |
| SGPL1 | 0.604532 | 1.00169 | 0.995308 | 1.008114 |  |
| SMPD1 | 0.983412 | 1.000058 | 0.994571 | 1.005576 |  |
| MOGAT2 | 0.055332 | 0.497438 | 0.243535 | 1.016054 |  |
| PLD4 | 0.026879 | 0.930035 | 0.872175 | 0.991733 |  |
| PLIN1 | 0.005006 | 1.119489 | 1.034636 | 1.2113 |  |
| PIP4K2C | 0.368719 | 1.001003 | 0.998817 | 1.003193 |  |
| MTMR10 | 0.014357 | 0.949676 | 0.911219 | 0.989755 |  |
| ACSF2 | 0.355146 | 0.996459 | 0.988995 | 1.00398 |  |
| DPEP2 | 0.003115 | 0.928497 | 0.883931 | 0.97531 |  |
| ACAA2 | 0.569723 | 0.998134 | 0.991728 | 1.004582 |  |
| GPX4 | 0.36743 | 0.999613 | 0.998771 | 1.000455 |  |
| MVD | 0.152324 | 1.007339 | 0.997303 | 1.017475 |  |
| GPD1 | 0.201448 | 0.980368 | 0.950994 | 1.01065 |  |
| ACER1 | 0.039936 | 0.189654 | 0.038826 | 0.926418 |  |
| ECI1 | 0.080477 | 1.005262 | 0.999364 | 1.011195 |  |
| HRASLS5 | 0.883601 | 1.001288 | 0.984178 | 1.018696 |  |
| RAB4A | 0.97043 | 0.999796 | 0.989061 | 1.010647 |  |
| ACOX2 | 0.056877 | 0.983182 | 0.966168 | 1.000496 |  |
| DEGS2 | 0.087309 | 0.98701 | 0.972326 | 1.001915 |  |
| MFSD2A | 0.037135 | 0.995138 | 0.990588 | 0.999709 |  |
| GSTM4 | 0.506103 | 0.987139 | 0.950184 | 1.025531 |  |
| PLA2G4F | 0.08193 | 0.983787 | 0.965834 | 1.002073 |  |
| INPP5D | 0.211038 | 0.990181 | 0.974988 | 1.005611 |  |
| B3GALNT1 | 0.00012 | 1.023883 | 1.011643 | 1.036271 |  |
| PLEKHA2 | 0.816538 | 0.998478 | 0.985711 | 1.01141 |  |
| AGPAT2 | 0.200838 | 0.999309 | 0.99825 | 1.000368 |  |
| FASN | 0.849082 | 1.000075 | 0.999302 | 1.000849 |  |
| FABP6 | 0.956864 | 0.999749 | 0.990679 | 1.008902 |  |
| GLB1 | 0.71286 | 1.000889 | 0.996164 | 1.005636 |  |
| FABP4 | 0.876726 | 0.999626 | 0.994907 | 1.004367 |  |
| HSD17B13 | 0.243809 | 0.992561 | 0.980176 | 1.005103 |  |
| ELOVL6 | 0.003514 | 1.020725 | 1.006761 | 1.034883 |  |
| PLA2G1B | 0.108783 | 0.997229 | 0.993853 | 1.000617 |  |
| MTM1 | 0.660097 | 0.9945 | 0.970354 | 1.019247 |  |
| PIK3CD | 0.044691 | 0.98368 | 0.968004 | 0.99961 |  |
| PTEN | 0.692024 | 0.997335 | 0.98425 | 1.010593 |  |
| CYP4F11 | 0.001985 | 1.008341 | 1.003047 | 1.013664 |  |
| CYP4F22 | 0.265042 | 1.011318 | 0.991499 | 1.031533 |  |
| ORMDL3 | 0.006125 | 0.994976 | 0.991398 | 0.998566 |  |
| MBOAT1 | 0.090187 | 0.992337 | 0.983546 | 1.001206 |  |
| CERS6 | 0.493734 | 1.004644 | 0.991401 | 1.018064 |  |
| SPTLC3 | 0.442798 | 0.990837 | 0.967816 | 1.014406 |  |
| STARD5 | 0.068533 | 0.786596 | 0.607545 | 1.018417 |  |
| PPP1CA | 0.189366 | 1.001032 | 0.999491 | 1.002576 |  |
| CYP7B1 | 0.328926 | 0.989668 | 0.969246 | 1.01052 |  |
| DHCR7 | 0.016409 | 1.003976 | 1.000727 | 1.007235 |  |
| LCLAT1 | 0.521739 | 1.00865 | 0.98242 | 1.03558 |  |
| ACSM6 | 0.284542 | 0.807843 | 0.546514 | 1.194133 |  |
| ARV1 | 0.64894 | 0.997862 | 0.988711 | 1.007099 |  |
| PHOSPHO1 | 0.659887 | 1.023595 | 0.922612 | 1.135631 |  |
| STARD6 | 0.414483 | 1.467072 | 0.584337 | 3.683324 |  |
| UGT8 | 0.486338 | 1.004583 | 0.991733 | 1.0176 |  |
| PTDSS2 | 0.801141 | 1.003119 | 0.979106 | 1.02772 |  |
| PCCA | 0.185244 | 1.011204 | 0.994672 | 1.028011 |  |
| GPX2 | 0.036307 | 1.000261 | 1.000017 | 1.000505 |  |
| SPHK1 | 2.87E-06 | 1.024827 | 1.014355 | 1.035407 |  |
| ACBD7 | 0.835028 | 0.995417 | 0.953304 | 1.039391 |  |
| HSD11B2 | 0.869584 | 0.999156 | 0.989132 | 1.009281 |  |
| LPCAT4 | 0.092312 | 1.004191 | 0.999313 | 1.009093 |  |
| PLA2G16 | 0.451523 | 1.000716 | 0.998854 | 1.002581 |  |
| ACSF3 | 0.291073 | 0.970751 | 0.918701 | 1.02575 |  |
| RUFY1 | 0.645271 | 1.002938 | 0.990488 | 1.015544 |  |
| SEC24C | 0.730779 | 0.998629 | 0.990857 | 1.006462 |  |
| ACER2 | 0.440251 | 0.991451 | 0.970068 | 1.013306 |  |
| ACOT4 | 0.308336 | 1.012799 | 0.988318 | 1.037886 |  |
| GBA | 0.27063 | 1.002719 | 0.997886 | 1.007576 |  |
| PNPLA2 | 0.530438 | 1.001467 | 0.996891 | 1.006064 |  |
| SLC25A20 | 0.538178 | 0.996121 | 0.983869 | 1.008525 |  |
| CPNE7 | 0.288485 | 0.992216 | 0.978001 | 1.006637 |  |
| ALOXE3 | 0.033819 | 1.156908 | 1.011206 | 1.323603 |  |
| ALOX12B | 0.002623 | 1.083793 | 1.028446 | 1.142118 |  |
| ALOX15B | 0.81247 | 0.999815 | 0.998285 | 1.001347 |  |
| PLD6 | 0.498644 | 0.989466 | 0.959573 | 1.020289 |  |
| TMEM86B | 0.062589 | 1.070367 | 0.996432 | 1.149787 |  |
| CYP8B1 | 0.681746 | 0.965972 | 0.81866 | 1.139792 |  |
| FAM73A | 0.92592 | 1.001159 | 0.977005 | 1.025911 |  |
| ARSJ | 0.261767 | 1.009655 | 0.992853 | 1.026741 |  |
| PITPNB | 0.053612 | 1.007302 | 0.999887 | 1.014773 |  |
| ACBD4 | 0.268075 | 0.985516 | 0.960396 | 1.011293 |  |
| ENPP7 | 0.776786 | 1.231687 | 0.291643 | 5.201751 |  |
| TNFAIP8L3 | 0.477247 | 1.009811 | 0.982987 | 1.037366 |  |
| ARSI | 0.042204 | 1.034371 | 1.001191 | 1.068652 |  |
| ACOT1 | 0.740822 | 0.994108 | 0.959897 | 1.029537 |  |
| PRKD1 | 0.418274 | 1.011264 | 0.984204 | 1.039069 |  |
| PLA2G6 | 0.985668 | 0.999805 | 0.978716 | 1.021348 |  |
| DGAT1 | 0.55593 | 1.001361 | 0.996838 | 1.005906 |  |
| INPP5J | 0.001028 | 0.923423 | 0.880527 | 0.968409 |  |
| TNFAIP8L1 | 0.729664 | 1.003325 | 0.984612 | 1.022393 |  |
| PCYT2 | 0.22316 | 1.009622 | 0.994186 | 1.025297 |  |
| CYP2R1 | 0.185152 | 0.974298 | 0.937486 | 1.012555 |  |
| PIP5K1C | 0.686639 | 1.003221 | 0.987667 | 1.019021 |  |
| GPAT2 | 0.236247 | 1.011206 | 0.99273 | 1.030026 |  |
| PPP1CC | 0.096029 | 1.005014 | 0.999112 | 1.01095 |  |
| INSIG1 | 0.392792 | 1.001889 | 0.997561 | 1.006236 |  |
| CYP4F3 | 0.016281 | 1.006174 | 1.001135 | 1.01124 |  |
| AKR1C1 | 0.912066 | 0.999969 | 0.999421 | 1.000518 |  |
| CIDEC | 0.000126 | 1.021057 | 1.01024 | 1.03199 |  |
| AGMO | 0.011293 | 1.064545 | 1.01426 | 1.117322 |  |
| PLA2G4E | 0.192823 | 0.982381 | 0.956448 | 1.009018 |  |
| PLA2G2A | 0.659444 | 0.99986 | 0.99924 | 1.000481 |  |
| ASAH2 | 0.507316 | 1.109437 | 0.816158 | 1.508104 |  |
| SUMO2 | 0.05151 | 1.002596 | 0.999983 | 1.005216 |  |
| PTPLAD2 | 0.097535 | 0.970236 | 0.93617 | 1.005542 |  |
| LIPI | 0.346057 | 0.789356 | 0.482608 | 1.291074 |  |
| AKR1C3 | 0.868677 | 1.000055 | 0.999405 | 1.000705 |  |
| THEM5 | 0.354668 | 1.003077 | 0.996572 | 1.009625 |  |
| PIK3R4 | 0.26192 | 1.00976 | 0.99277 | 1.02704 |  |
| SPTSSB | 0.723925 | 1.00175 | 0.992078 | 1.011516 |  |
| GM2A | 0.902807 | 1.000192 | 0.997114 | 1.00328 |  |
| ACSL5 | 0.049671 | 0.998649 | 0.997302 | 0.999998 |  |
| FITM2 | 0.17118 | 0.987108 | 0.968932 | 1.005625 |  |
| SLC22A5 | 0.318647 | 1.039771 | 0.96305 | 1.122603 |  |
| FAR1 | 0.383969 | 0.994666 | 0.982763 | 1.006714 |  |
| PSAP | 0.416719 | 0.999928 | 0.999755 | 1.000101 |  |
| ELOVL2 | 0.011125 | 1.028456 | 1.006417 | 1.050978 |  |
| HSD17B11 | 0.211209 | 0.998998 | 0.997429 | 1.000569 |  |
| AKR1C4 | 0.975137 | 1.000166 | 0.989777 | 1.010664 |  |
| ECI2 | 0.845026 | 1.000846 | 0.992397 | 1.009366 |  |
| OXCT2 | 0.167954 | 0.889068 | 0.752223 | 1.050808 |  |
| GK | 0.375489 | 0.995493 | 0.985596 | 1.005488 |  |
| INPP5F | 0.237055 | 0.990394 | 0.974674 | 1.006368 |  |
| SGMS1 | 0.858108 | 0.999197 | 0.99044 | 1.008032 |  |
| NEU4 | 0.094473 | 1.083278 | 0.986332 | 1.189753 |  |
| AWAT1 | 0.615962 | 0.959095 | 0.814677 | 1.129115 |  |
| HSD17B8 | 0.248006 | 0.996929 | 0.99174 | 1.002145 |  |
| AGPAT1 | 0.279903 | 1.003322 | 0.997305 | 1.009375 |  |
| SLC44A4 | 0.324803 | 0.999364 | 0.998099 | 1.000631 |  |
| NEU1 | 0.157904 | 0.997644 | 0.994382 | 1.000916 |  |
| CSNK2B | 0.507504 | 1.002392 | 0.995335 | 1.009499 |  |
| CPT1B | 0.413819 | 0.967837 | 0.894851 | 1.046777 |  |
| ARSH | 0.112561 | 1.085345 | 0.980924 | 1.200882 |  |
| ACOT6 | 0.897538 | 1.05352 | 0.476442 | 2.329567 |  |
| PPAPDC2 | 0.61723 | 0.994593 | 0.973668 | 1.015967 |  |
| PTPLB | 0.007117 | 1.006355 | 1.001723 | 1.011008 |  |
| SACM1L | 0.144616 | 0.986319 | 0.968232 | 1.004744 |  |
| LTC4S | 0.154042 | 0.315148 | 0.064411 | 1.541953 |  |
| HEXA | 0.399954 | 0.994795 | 0.982779 | 1.006958 |  |
| PPP1CB | 0.552129 | 1.000624 | 0.998568 | 1.002685 |  |
| NUDT19 | 0.072082 | 1.005199 | 0.999535 | 1.010896 |  |
| CPNE1 | 0.552102 | 1.00063 | 0.998555 | 1.002709 |  |
| STARD10 | 0.349576 | 0.998899 | 0.996596 | 1.001207 |  |
| PPT2 | 6.12E-05 | 1.053697 | 1.027089 | 1.080995 |  |
| CERS1 | 0.398492 | 1.064276 | 0.920983 | 1.229862 |  |
| CPTP | 0.165814 | 1.005161 | 0.997869 | 1.012506 |  |
| AKR1B15 | 0.392924 | 1.006584 | 0.991543 | 1.021853 |  |
| CYP21A2 | 0.727729 | 0.982478 | 0.88941 | 1.085284 |  |
| GPX1 | 0.744987 | 1.000068 | 0.999657 | 1.00048 |  |
| ACAD11 | 0.260413 | 0.718404 | 0.403858 | 1.277937 |  |
| PISD | 0.953956 | 1.000433 | 0.985839 | 1.015243 |  |
| PI4KA | 0.789274 | 0.998178 | 0.984916 | 1.011618 |  |
| AMACR | 0.215037 | 0.975406 | 0.937755 | 1.014569 |  |
| DECR2 | 0.35399 | 1.00647 | 0.992838 | 1.020288 |  |
| PLA2G4B | 0.034672 | 0.656393 | 0.444123 | 0.970118 |  |
| FDX1L | 0.670831 | 0.988167 | 0.935386 | 1.043927 |  |
| PIK3R6 | 0.615535 | 0.984523 | 0.926376 | 1.04632 |  |
| PIP4K2B | 0.882289 | 0.999356 | 0.990871 | 1.007914 |  |
| SRD5A2 | 0.785374 | 1.003929 | 0.975994 | 1.032663 |  |
| ACACA | 0.698959 | 0.997599 | 0.985519 | 1.009827 |  |
| ZNF638 | 0.70602 | 0.997619 | 0.985338 | 1.010053 |  |
| KLF5 | 0.005095 | 1.002249 | 1.000675 | 1.003825 |  |
| TGFB1 | 0.2731 | 1.001777 | 0.998602 | 1.004961 |  |
| NFKB1 | 0.466979 | 1.003493 | 0.99411 | 1.012964 |  |
| CCND3 | 0.815809 | 0.999712 | 0.997295 | 1.002136 |  |
| EGR2 | 0.05246 | 0.984622 | 0.969321 | 1.000165 |  |
| PCK1 | 0.038052 | 1.01036 | 1.000568 | 1.020249 |  |
| WNT1 | 0.001116 | 1.168086 | 1.0639 | 1.282474 |  |
| CDK4 | 0.902303 | 1.000125 | 0.99813 | 1.002125 |  |
| KLF4 | 0.019905 | 1.005397 | 1.000852 | 1.009964 |  |
| EBF1 | 0.349264 | 1.035075 | 0.96299 | 1.112555 |  |
| WNT10B | 0.526908 | 1.032354 | 0.935391 | 1.139368 |  |
| CEBPB | 0.002128 | 1.002324 | 1.00084 | 1.003809 |  |
| RELA | 0.007665 | 1.00994 | 1.002624 | 1.017309 |  |
| LEP | 0.394912 | 1.007058 | 0.990872 | 1.023509 |  |
| LPL | 0.606167 | 0.999502 | 0.997609 | 1.001398 |  |
| ZNF467 | 0.459738 | 1.005204 | 0.991466 | 1.019133 |  |
| SLC2A4 | 0.188778 | 0.85127 | 0.669533 | 1.082338 |  |
| NR2F2 | 0.055115 | 1.004765 | 0.999896 | 1.009658 |  |
| CEBPD | 0.600371 | 1.000349 | 0.999043 | 1.001657 |  |
| TNF | 0.363813 | 0.985264 | 0.954196 | 1.017344 |  |
| CEBPA | 0.974095 | 0.999936 | 0.996107 | 1.003781 |  |
| DGKZ | 0.138804 | 1.015983 | 0.994874 | 1.03754 |  |
| DGKE | 0.399545 | 0.970327 | 0.904654 | 1.040767 |  |
| DGKD | 0.435012 | 0.995482 | 0.98423 | 1.006864 |  |
| DGKH | 0.56421 | 0.990374 | 0.958342 | 1.023477 |  |
| JMJD7-PLA2G4B | 0.030589 | 0.903325 | 0.823798 | 0.990528 |  |
| PLA2G12B | 0.351738 | 0.996117 | 0.987994 | 1.004307 |  |
| LYPLA1 | 0.406379 | 1.000954 | 0.998703 | 1.003211 |  |
| ADPRM | 0.830357 | 0.996139 | 0.961505 | 1.032021 |  |
| DGKQ | 0.856504 | 0.998654 | 0.984176 | 1.013345 |  |
| DGKB | 0.800232 | 1.014373 | 0.908223 | 1.132931 |  |
| DGKG | 0.127688 | 1.068641 | 0.981149 | 1.163935 |  |
| DGKA | 0.206393 | 1.012561 | 0.993144 | 1.032357 |  |
| DGKI | 0.176265 | 1.188593 | 0.92531 | 1.526789 |  |
| LYPLA2 | 0.65703 | 1.000554 | 0.998111 | 1.003002 |  |
| PLA2G2C | 0.571245 | 0.932092 | 0.730703 | 1.188986 |  |
| LCAT | 0.214637 | 0.983511 | 0.958021 | 1.009681 |  |
