# Supplementary material for: A Lipid Metabolism-Based Seven-Gene Signature Correlates with the Clinical Outcome of Lung Adenocarcinoma
Source: J Oncol. 2022 Feb 11;2022:9913206. doi: 10.1155/2022/9913206 (PMC8856807; doi:10.1155/2022/9913206)
Supplement: Supplementary Materials — Supplementary Figure 1: (A) NMF rank survey of cophenetic, RSS, and dispersion; (B) distribution of clinicopathological parameters in the three subtypes; (C) proportions of B cell, CD4+T cell, CD8+T cell, neutrophil, macrophage, and dendritic cell (DC) in the three subtypes; and (D) distribution of the tumor immune estimation resource, tumor stromal, immune, and estimate scores in the three subtypes. Supplementary Figure 2: (A) Hierarchical clustering for identification of samples with outliers; (B and C) analysis of network topology for various soft-thresholding powers; (D) the Venn diagrams of the overlapping genes among the DEGs in each molecular subtype; (E) enriched top 20 KEGG pathways of overlapping genes; and (F–H) enriched top 20 gene oncology (GO) cellular component, molecular function, and biological process of overlapping genes. The color from red to blue represents the significance of P value; the redder color represents smaller P value; the dot size represents the number of genes enriched into the pathway; larger number represents a larger value. Supplementary Figure 3: (A) Confidence intervals of each lambda; (B) trajectory change of each independent variable, the X-axis represents the log value of the independent variable lambda and the Y-axis represents the coefficient of the independent variable; (C-I) Kaplan–Meier survival analysis of overall survival for patients with high or low expression of CHRDL1 (C), GAPDH (D), GNPNAT1 (E), HTATIP2 (F), MFI2 (G), PKP2 (H), and RGS20 (I) in the training set. Supplementary Figure 4. Forest plot of the univariate (A) and multivariate (B) Cox regression analyses in the TCGA LUAD data set. Supplementary Figure 5. Kaplan–Meier and ROC curve survival analysis of the gene signature of Shukla et al. (A), Li et al. (B), Yue et al. (C), and Liu et al. (D). [file 9913206.f1.zip › 9913206.f1/Supplementary Table 4.docx]

| **Supplementary Table 4.** Overlapping DEGs significantly associated with patient with LUAD in training set | | | | |
| --- | --- | --- | --- | --- |
| Symbol | P value | Harzard ratio | Low 95%CI | High 95%CI |
| PGD | 0.006888982 | 1.000659393 | 1.000181071 | 1.001137944 |
| AGMAT | 0.002318297 | 1.052003805 | 1.018241124 | 1.086885984 |
| RAB3B | 0.00445057 | 1.017031089 | 1.005264547 | 1.028935358 |
| LRP8 | 0.005617284 | 1.020903762 | 1.006064649 | 1.035961746 |
| UCK2 | 2.03E-07 | 1.021483137 | 1.013326348 | 1.029705584 |
| ACOXL | 0.003130382 | 0.913003267 | 0.859511259 | 0.969824371 |
| PFN2 | 0.002257072 | 1.002186509 | 1.000782789 | 1.003592197 |
| PPP2R2C | 1.14E-06 | 1.03267663 | 1.019385561 | 1.046140992 |
| C1QTNF7 | 0.002716349 | 0.874946607 | 0.80177649 | 0.954794228 |
| SLC34A2 | 0.000563531 | 0.999738158 | 0.999589374 | 0.999886965 |
| NEIL3 | 0.002689516 | 1.020858055 | 1.007187591 | 1.034714067 |
| C7 | 0.005917663 | 0.995373492 | 0.992091935 | 0.998665904 |
| STC2 | 1.02E-05 | 1.018145217 | 1.010044676 | 1.026310723 |
| KIAA0319 | 0.009498035 | 1.019455723 | 1.00471856 | 1.03440905 |
| GPR116 | 0.005367152 | 0.997860891 | 0.996357752 | 0.999366297 |
| GCLC | 0.001748986 | 1.002057823 | 1.000768683 | 1.003348624 |
| POPDC3 | 0.000462694 | 1.022830603 | 1.009987605 | 1.035836913 |
| AKR1B10 | 0.007961322 | 1.00026195 | 1.000068473 | 1.000455464 |
| PBK | 0.000205988 | 1.012166127 | 1.005723225 | 1.018650305 |
| GSR | 0.006900845 | 1.001469739 | 1.000403251 | 1.002537364 |
| RGS20 | 2.18E-07 | 1.083166404 | 1.050937336 | 1.11638384 |
| FAM189A2 | 0.001467423 | 0.96220812 | 0.9396377 | 0.98532069 |
| MAMDC2 | 0.008108894 | 0.972432398 | 0.952514649 | 0.992766642 |
| TXN | 0.003519257 | 1.000435136 | 1.000142875 | 1.000727483 |
| PTGR1 | 0.007743346 | 1.001369092 | 1.000361283 | 1.002377916 |
| ABCC2 | 2.13E-05 | 1.006997616 | 1.003765221 | 1.01024042 |
| GFRA1 | 0.003322261 | 0.843571971 | 0.753022463 | 0.945009883 |
| HTATIP2 | 4.26E-05 | 1.006030009 | 1.00313773 | 1.008930628 |
| TXNRD1 | 1.16E-05 | 1.000752852 | 1.000416303 | 1.001089515 |
| OIP5 | 0.000103992 | 1.02805049 | 1.013787891 | 1.042513744 |
| SLC7A5 | 1.62E-06 | 1.002711908 | 1.001602733 | 1.003822311 |
| TRIM16 | 0.000176188 | 1.018584439 | 1.008830845 | 1.028432332 |
| CBX2 | 0.009401634 | 1.013226004 | 1.003228588 | 1.023323047 |
| CYP4F3 | 0.009438048 | 1.006626056 | 1.001618928 | 1.011658214 |
| CYP4F11 | 0.000303491 | 1.009679292 | 1.00441593 | 1.014970235 |
| SRXN1 | 0.007125555 | 1.019092028 | 1.005150446 | 1.033226983 |
| SUSD2 | 0.009822812 | 0.998452668 | 0.997279708 | 0.999627007 |
| PIR | 0.00071337 | 1.008207109 | 1.003446042 | 1.012990766 |
| PRDM16 | 0.009313709 | 0.960054921 | 0.931004816 | 0.990011475 |
| AUNIP | 0.006492101 | 1.028368752 | 1.00786089 | 1.049293907 |
| CLSPN | 0.00389615 | 1.025607144 | 1.008148868 | 1.043367746 |
| RSPO1 | 0.000733344 | 0.582438597 | 0.42559499 | 0.797083442 |
| CDCA8 | 0.003501272 | 1.006478122 | 1.002125132 | 1.010850019 |
| SLC2A1 | 1.04E-10 | 1.002671454 | 1.001860305 | 1.003483259 |
| CDC20 | 0.000641505 | 1.003565406 | 1.001516628 | 1.005618375 |
| KIF2C | 0.000968184 | 1.008116419 | 1.003287371 | 1.01296871 |
| ORC1 | 0.000239374 | 1.027244459 | 1.012616796 | 1.042083424 |
| DEPDC1 | 0.000583464 | 1.027959942 | 1.011931371 | 1.044242398 |
| SLC16A1 | 0.002514012 | 1.003979015 | 1.001396265 | 1.006568426 |
| FAM72B | 0.000271419 | 1.211723003 | 1.092720038 | 1.343686017 |
| FAM72D | 0.006618771 | 1.192169334 | 1.050118632 | 1.353435392 |
| CKS1B | 0.001093816 | 1.005896105 | 1.002352775 | 1.009451961 |
| PRG4 | 0.00943415 | 0.966834747 | 0.942525035 | 0.991771458 |
| ASPM | 0.003583253 | 1.018468802 | 1.006003912 | 1.031088138 |
| KIF14 | 6.70E-05 | 1.042646136 | 1.021458065 | 1.064273712 |
| UBE2T | 0.002365225 | 1.004084306 | 1.001449207 | 1.006726339 |
| FAM72A | 9.52E-06 | 1.414773367 | 1.213344267 | 1.649642013 |
| NEK2 | 7.02E-07 | 1.019703707 | 1.011873151 | 1.027594861 |
| DTL | 7.05E-06 | 1.03281696 | 1.018367885 | 1.047471044 |
| CENPF | 0.000186579 | 1.014179758 | 1.006716993 | 1.021697845 |
| EXO1 | 5.00E-06 | 1.024953835 | 1.014164048 | 1.035858415 |
| RRM2 | 2.02E-06 | 1.008224996 | 1.004823745 | 1.011637759 |
| MTHFD2 | 0.003300148 | 1.004470733 | 1.001486363 | 1.007463996 |
| NCAPH | 0.000943575 | 1.012572806 | 1.005101932 | 1.02009921 |
| BUB1 | 0.000410834 | 1.013923491 | 1.00617559 | 1.021731053 |
| CKAP2L | 5.56E-05 | 1.034776003 | 1.017717863 | 1.052120058 |
| CD302 | 0.00146562 | 0.970694358 | 0.95306922 | 0.988645438 |
| SCN7A | 0.007239668 | 0.961266956 | 0.933949849 | 0.989383062 |
| SPC25 | 0.000192133 | 1.026120598 | 1.012308171 | 1.040121488 |
| SGOL2 | 0.000109881 | 1.043301485 | 1.021128832 | 1.065955592 |
| HJURP | 1.61E-05 | 1.018248463 | 1.009916075 | 1.026649597 |
| SGOL1 | 0.000450084 | 1.05619915 | 1.024428997 | 1.088954578 |
| CLEC3B | 0.00071863 | 0.986196596 | 0.978285375 | 0.994171793 |
| CDC25A | 0.000365355 | 1.039787226 | 1.017714428 | 1.062338752 |
| KIAA1524 | 0.006554926 | 1.022081701 | 1.006113831 | 1.038302996 |
| PLOD2 | 0.000351083 | 1.003227402 | 1.001456402 | 1.005001534 |
| ECT2 | 4.19E-07 | 1.010305066 | 1.006300873 | 1.014325191 |
| MFI2 | 1.91E-08 | 1.014618569 | 1.009496317 | 1.019766812 |
| NCAPG | 0.000461942 | 1.017112354 | 1.007499063 | 1.026817373 |
| ADH1B | 0.002115531 | 0.988489382 | 0.981218404 | 0.995814239 |
| CENPE | 0.001273629 | 1.039418618 | 1.015257648 | 1.064154569 |
| MAD2L1 | 0.001071073 | 1.017905032 | 1.007138833 | 1.028786321 |
| CCNA2 | 2.51E-05 | 1.009522371 | 1.005082316 | 1.01398204 |
| PLK4 | 0.003579066 | 1.032484503 | 1.010514199 | 1.054932478 |
| MND1 | 0.004470334 | 1.029969998 | 1.009213478 | 1.051153419 |
| CENPU | 0.001357609 | 1.013280224 | 1.005134366 | 1.021492097 |
| TRIP13 | 0.006163859 | 1.00825498 | 1.002340868 | 1.014203986 |
| C5orf38 | 0.009072075 | 0.985303403 | 0.974406455 | 0.996322213 |
| DEPDC1B | 0.006519652 | 1.021576243 | 1.005985108 | 1.037409014 |
| CCNB1 | 3.49E-06 | 1.005625151 | 1.003245031 | 1.008010918 |
| KIF20A | 5.15E-06 | 1.01703834 | 1.009677729 | 1.024452609 |
| CDC25C | 1.04E-05 | 1.056087541 | 1.03077989 | 1.082016544 |
| HMMR | 2.19E-05 | 1.020862672 | 1.011176356 | 1.030641777 |
| PHACTR1 | 0.004545392 | 0.935013293 | 0.892609063 | 0.979431975 |
| KIFC1 | 0.009047471 | 1.005207701 | 1.001294862 | 1.00913583 |
| HMGA1 | 3.61E-06 | 1.000680337 | 1.000392443 | 1.000968314 |
| TTK | 0.003661936 | 1.02137985 | 1.006910055 | 1.036057582 |
| MTFR2 | 0.005407568 | 1.035717431 | 1.010421037 | 1.061647133 |
| STEAP1B | 0.000257276 | 1.028347934 | 1.013047698 | 1.043879252 |
| IGF2BP3 | 0.000817757 | 1.018000348 | 1.007419709 | 1.028692113 |
| HOXA1 | 0.003138553 | 1.017249495 | 1.005770989 | 1.028859001 |
| ANLN | 1.34E-10 | 1.011732417 | 1.008137765 | 1.015339885 |
| DLC1 | 0.009726675 | 0.989830177 | 0.982189515 | 0.997530277 |
| STC1 | 1.32E-05 | 1.003191608 | 1.001754497 | 1.004630781 |
| CDCA2 | 4.32E-05 | 1.046876525 | 1.024141872 | 1.070115859 |
| ESCO2 | 0.000693741 | 1.111617079 | 1.045687001 | 1.181704018 |
| GINS4 | 0.004982191 | 1.030668838 | 1.009166144 | 1.052629698 |
| MCM4 | 0.00019521 | 1.005855502 | 1.002770394 | 1.008950103 |
| MELK | 0.001130613 | 1.011551723 | 1.00458186 | 1.018569943 |
| SAPCD2 | 0.002391758 | 1.016422312 | 1.005792379 | 1.02716459 |
| PFKP | 0.000255629 | 1.00235541 | 1.001092188 | 1.003620226 |
| ZWINT | 0.002385094 | 1.004859549 | 1.001721254 | 1.008007675 |
| CDK1 | 0.001104403 | 1.007349316 | 1.002927703 | 1.011790423 |
| KIF11 | 0.000100168 | 1.014171487 | 1.007006641 | 1.021387311 |
| CEP55 | 0.00023606 | 1.010035912 | 1.004673858 | 1.015426583 |
| HPSE2 | 0.004331 | 0.698842311 | 0.54634866 | 0.893899101 |
| MKI67 | 2.40E-05 | 1.012738153 | 1.006807256 | 1.018703989 |
| ADM | 1.27E-05 | 1.007431644 | 1.004087951 | 1.010786471 |
| KIF18A | 1.60E-07 | 1.063589684 | 1.039348848 | 1.088395893 |
| CDCA5 | 1.34E-05 | 1.01369615 | 1.007507137 | 1.019923181 |
| MTL5 | 0.000183865 | 1.051357108 | 1.024124089 | 1.079314294 |
| CHEK1 | 0.000252952 | 1.024862964 | 1.01146998 | 1.038433285 |
| ADAMTS8 | 0.00461721 | 0.932560094 | 0.888577335 | 0.978719909 |
| FOXM1 | 1.43E-05 | 1.009227949 | 1.005049162 | 1.013424111 |
| GAPDH | 1.16E-09 | 1.000252358 | 1.000171083 | 1.000333639 |
| CDCA3 | 0.0001249 | 1.043801302 | 1.0211887 | 1.066914623 |
| ARNTL2 | 8.04E-10 | 1.014305604 | 1.009720106 | 1.018911926 |
| PKP2 | 1.44E-06 | 1.01611407 | 1.009529097 | 1.022741996 |
| RACGAP1 | 0.001360856 | 1.009193555 | 1.00355759 | 1.014861171 |
| ESPL1 | 0.003775308 | 1.027941459 | 1.008949344 | 1.047291075 |
| E2F7 | 1.36E-10 | 1.101642887 | 1.069565637 | 1.134682164 |
| SKA3 | 5.70E-05 | 1.03344818 | 1.017022957 | 1.050138675 |
| DIAPH3 | 0.000132699 | 1.058401706 | 1.028033969 | 1.089666493 |
| RNASE1 | 0.009631855 | 0.999812173 | 0.999669993 | 0.999954374 |
| SFTA3 | 0.002657169 | 0.996981897 | 0.995018195 | 0.998949475 |
| PYGL | 0.000266505 | 1.006680793 | 1.003083756 | 1.010290728 |
| ERO1L | 1.01E-08 | 1.004008621 | 1.00263556 | 1.005383562 |
| GNPNAT1 | 2.79E-12 | 1.013681139 | 1.009825212 | 1.017551789 |
| CDKN3 | 0.000222078 | 1.013993769 | 1.006541681 | 1.021501031 |
| DLGAP5 | 1.30E-05 | 1.013204928 | 1.007246818 | 1.019198282 |
| CDCA4 | 9.94E-06 | 1.014001999 | 1.007766871 | 1.020275704 |
| ARHGAP11A | 1.10E-05 | 1.02642186 | 1.014559341 | 1.038423078 |
| BUB1B | 0.00029616 | 1.017633897 | 1.008044585 | 1.027314431 |
| CASC5 | 0.000616222 | 1.054275617 | 1.022859969 | 1.08665615 |
| RAD51 | 0.000256555 | 1.032432069 | 1.014914949 | 1.050251529 |
| NUSAP1 | 0.000769485 | 1.006782931 | 1.002824889 | 1.010756595 |
| CCNB2 | 0.00325395 | 1.005868808 | 1.001956073 | 1.009796823 |
| KIAA0101 | 0.002923555 | 1.01132725 | 1.003852059 | 1.018858106 |
| KIF23 | 0.001155248 | 1.017339604 | 1.006846077 | 1.027942496 |
| TICRR | 6.75E-05 | 1.090557038 | 1.04503646 | 1.138060441 |
| BLM | 0.001274921 | 1.03356392 | 1.013012177 | 1.054532612 |
| PRC1 | 2.14E-06 | 1.015518191 | 1.009072242 | 1.022005317 |
| PKMYT1 | 0.002950509 | 1.03044296 | 1.010269898 | 1.051018838 |
| PLK1 | 1.94E-06 | 1.016621202 | 1.009742966 | 1.023546291 |
| SHCBP1 | 1.74E-05 | 1.033802185 | 1.018240285 | 1.049601919 |
| CENPN | 0.001435732 | 1.029130483 | 1.011118764 | 1.047463057 |
| GSG2 | 0.000295335 | 1.076157346 | 1.03422331 | 1.119791656 |
| GGT6 | 0.008099995 | 0.985621243 | 0.975111366 | 0.996244398 |
| FAM64A | 0.004681323 | 1.016861076 | 1.00514619 | 1.028712498 |
| MFAP4 | 0.0072763 | 0.998011799 | 0.996562394 | 0.999463313 |
| SPAG5 | 0.006798625 | 1.007597684 | 1.002090037 | 1.013135602 |
| CDC6 | 0.007376126 | 1.008882488 | 1.002377219 | 1.015429976 |
| EME1 | 0.00618135 | 1.052621452 | 1.014679624 | 1.091982035 |
| PRR11 | 2.49E-05 | 1.020951499 | 1.011156933 | 1.03084094 |
| KPNA2 | 4.51E-06 | 1.003211622 | 1.001837849 | 1.00458728 |
| TK1 | 3.75E-05 | 1.003051405 | 1.001599507 | 1.004505407 |
| BIRC5 | 0.000239159 | 1.007470316 | 1.003477782 | 1.011478736 |
| NDC80 | 0.000344268 | 1.014625273 | 1.006590845 | 1.022723829 |
| SKA1 | 0.001661892 | 1.023153243 | 1.008661153 | 1.03785355 |
| PMAIP1 | 0.00711047 | 1.005312657 | 1.001441349 | 1.00919893 |
| UHRF1 | 0.003943239 | 1.0184181 | 1.00585885 | 1.031134166 |
| ASF1B | 0.002912735 | 1.007425652 | 1.002530331 | 1.012344876 |
| CPAMD8 | 0.008609964 | 0.979618901 | 0.964684848 | 0.994784145 |
| ZNF540 | 0.001080447 | 0.761199674 | 0.646313426 | 0.896507671 |
| TNNT1 | 0.000639799 | 1.004080343 | 1.00173591 | 1.006430263 |
| UBE2S | 4.69E-05 | 1.010475445 | 1.005418009 | 1.015558321 |
| TPX2 | 0.000146068 | 1.003341183 | 1.001615415 | 1.005069925 |
| FAM83D | 0.004091171 | 1.010025037 | 1.003170463 | 1.016926447 |
| UBE2C | 0.003829426 | 1.001710962 | 1.000551043 | 1.002872225 |
| AURKA | 3.82E-05 | 1.009396377 | 1.004913139 | 1.013899616 |
| SEC14L6 | 0.004716058 | 0.965564174 | 0.94237869 | 0.989320093 |
| GTSE1 | 0.000223994 | 1.030638966 | 1.014251113 | 1.047291606 |
| FIGF | 0.002977684 | 0.968142696 | 0.947677519 | 0.989049821 |
| KIF4A | 4.58E-06 | 1.021225352 | 1.012094252 | 1.030438832 |
| ERCC6L | 0.001426205 | 1.059704022 | 1.022604105 | 1.098149919 |
| CHRDL1 | 0.00209456 | 0.981517425 | 0.969921109 | 0.993252387 |
